# Survey statistics of automated segmentations applied to optical imaging of mammalian cells

**DOI:** 10.1186/s12859-015-0762-2

**Published:** 2015-10-15

**Authors:** Peter Bajcsy, Antonio Cardone, Joe Chalfoun, Michael Halter, Derek Juba, Marcin Kociolek, Michael Majurski, Adele Peskin, Carl Simon, Mylene Simon, Antoine Vandecreme, Mary Brady

**Affiliations:** 1 Information Technology Laboratory, National Institute of Standards and Technology, Gaithersburg, USA; 2 Material Measurement Laboratory, National Institute of Standards and Technology, Gaithersburg, USA; 3 Lodz University of Technology, Lodz, Poland

**Keywords:** Cellular measurements, Cell segmentation, Segmented objects, Segmentation evaluation, Accelerated execution of segmentation for high-throughput biological application

## Abstract

**Background:**

The goal of this survey paper is to overview cellular measurements using optical microscopy imaging followed by automated image segmentation. The cellular measurements of primary interest are taken from mammalian cells and their components. They are denoted as two- or three-dimensional (2D or 3D) image objects of biological interest. In our applications, such cellular measurements are important for understanding cell phenomena, such as cell counts, cell-scaffold interactions, cell colony growth rates, or cell pluripotency stability, as well as for establishing quality metrics for stem cell therapies. In this context, this survey paper is focused on automated segmentation as a software-based measurement leading to quantitative cellular measurements.

**Methods:**

We define the scope of this survey and a classification schema first. Next, all found and manually filteredpublications are classified according to the main categories: (1) objects of interests (or objects to be segmented), (2) imaging modalities, (3) digital data axes, (4) segmentation algorithms, (5) segmentation evaluations, (6) computational hardware platforms used for segmentation acceleration, and (7) object (cellular) measurements. Finally, all classified papers are converted programmatically into a set of hyperlinked web pages with occurrence and co-occurrence statistics of assigned categories.

**Results:**

The survey paper presents to a reader: (a) the state-of-the-art overview of published papers about automated segmentation applied to optical microscopy imaging of mammalian cells, (b) a classification of segmentation aspects in the context of cell optical imaging, (c) histogram and co-occurrence summary statistics about cellular measurements, segmentations, segmented objects, segmentation evaluations, and the use of computational platforms for accelerating segmentation execution, and (d) open research problems to pursue.

**Conclusions:**

The novel contributions of this survey paper are: (1) a new type of classification of cellular measurements and automated segmentation, (2) statistics about the published literature, and (3) a web hyperlinked interface to classification statistics of the surveyed papers at https://isg.nist.gov/deepzoomweb/resources/survey/index.html.

## Background

Segmentation is one of the fundamental digital image processing operations. It is used ubiquitously across all scientific and industrial fields where imaging has become the qualitative observation and quantitative measurement method. Segmentation design, evaluation, and computational scalability can be daunting for cell biologists because of a plethora of segmentation publications scattered across many fields with reported segmentation choices that are highly dependent on scientific domain specific image content. Thus, the goal of this survey paper is to overview automated image segmentations used for cellular measurements in biology.

In quantitative image-based cell biology, cellular measurements are primarily derived from detected objects using image segmentation methods. In order to report statistically significant results for any hypothesis or task, cellular measurements have to be taken from a large number of images. This requires automated segmentation which includes algorithm design, evaluation, and computational scalability in high-throughput applications. This survey is motivated by the need to provide a statistics-based guideline for cell biologists to map their cellular measurement tasks to the frequently used segmentation choices.

The large number of publications reporting on both the omnipresent image segmentation problem and cell biology problems using image-based cellular measurements was narrowed down by adding more specific cell biology criteria and considering recent publications dated from the year 2000 until the present. While general survey papers are cited without any date constraints to provide references to segmentation fundamentals, statistics-based guidelines are reported for selected published papers that focus on optical microscopy imaging of mammalian cells and that utilize three-dimensional (3D) image cubes consisting of X-Y-Time or X-Y-Z dimensions (or X-Y-Z over time). Although there are many promising optical microscopy imaging modalities, we have primarily focused on the conventional phase contrast, differential interference contrast (DIC), confocal laser scanning, and fluorescent and dark/bright field modalities. In the space of mammalian cells and their cellular measurements, we included publications reporting *in vitro* cell cultures. The goal of such cellular measurements is to understand the spectrum of biological and medical problems in the realm of stem cell therapies and regenerative medicine, or cancer research and drug design. We introduce first the basic motivations behind cellular measurements via microscopy imaging and segmentation. Next we describe the types of results that come from image segmentation and the requirements that are imposed on segmentation methods.

### Motivation

We address three motivational questions behind this survey: (1) why is quantitative cell imaging important for cell biology; (2) why is segmentation critical to cellular measurements; and (3) why is automation of segmentation important to cell biology research? We analyze image segmentation and cellular characterization as software-based cellular measurements that are applied to images of mammalian cells.

First, cell research has its unique role in understanding living biological systems and developing next generation regenerative medicine and stem cell therapies for repairing diseases at the cellular level. Live cell imaging and 3D cell imaging play an important role in both basic science and drug discovery at the levels of a single cell and its components, as well as at the levels of tissues and organs [1]. While qualitative cell imaging is commonly used to explore complex cell biological phenomena, quantitative cell imaging is less frequently used because of the additional complexity associated with qualifying the quantitative aspects of the instrumentation, and the need for software-based analysis. If quantitative cell imaging is enabled then a wide range of applications can benefit from high statistical confidence in cellular measurements at a wide range of length scales. For example, quantitative cell imaging is potentially a powerful tool for qualifying cell therapy products such as those that can cure macular degeneration, the leading cause of blindness in adults (7 million US patients, gross domestic product loss $30 billion [2]). On the research side, quantitative cell imaging is needed to improve our understanding of complex cell phenomena, such as cell-scaffold interactions, and cell colony behavior such as pluripotency stability, and is especially powerful when these phenomena can be studied in live cells dynamically.

Second, the segmentation of a variety of cell microscopy image types is a necessary step to isolate an object of interest from its background for cellular measurements. At a very low level, segmentation is a partition of an image into connected groups of pixels that have semantic meaning. Mammalian cell segmentation methods can be found in literature that focus on biological and medical image informatics. They aim to improve the efficiency, accuracy, usability, and reliability of medical imaging services within the healthcare enterprise [3]. Segmentation methods also become a part of quantitative techniques for probing cellular structure and dynamics, and for cell-based screens [4]. Cellular measurement without image segmentation would be limited to statistics of either a portion of a cell (i.e., portion of a cell interior covered by one field of view) or mixture of a cell and its background. Thus, accurate and efficient segmentation becomes critical for cellular measurements.

Third, with the advancements in cell microscopy imaging and the increasing quantity of images, the automation of segmentation has gained importance not only for industrial applications but also for basic research. The benefits of automation can be quantified in terms of its cost, efficiency, and reproducibility of image segmentation per cell. The benefits motivate the design of automated segmentations while maximizing their accuracy. However, with automation comes a slew of questions for cell biologists about design and evaluations of accuracy, precision, and computational efficiency.

**Fig. 1:**
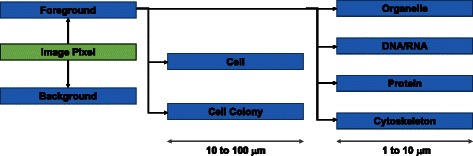
Segmentation labels ranging from generic (foreground, background) to cell specific objects relevant to diffraction-limited microscopy (DNA/RNA, protein, organelle, or cytoskeleton)

Image segmentation results are objects of interest to cell biologists that can be described by semantically meaningful terms in cell biology and can also be characterized by spectral intensity, shape, motion, or textural measurements from acquired images. Fig. 1 illustrates generic and cell specific labels assigned to a 2D image pixel (or 3D image voxel) during segmentation. Specific semantic labels depend on the type of experiment. For instance, the stain choice in an experimental design followed by imaging modality and segmentation method determines a semantic label of a segmentation result. It is also common to incorporate *a priori* knowledge about cells to obtain semantically meaningful segmentation results. For example, cell connectivity defines segmentation results at the image level to be connected sets of 2D pixels or 3D voxels.

### Segmentation results and imaging measurement pipeline

Given a connected set of 2D pixels or 3D voxels as a segmentation result, one can obtain cellular measurements about (1) motility of cells, (2) cell and organelle morphology, (3) cell proliferation, (4) location and spatial distribution of biomarkers in cells, (5) populations of cells with multiple phenotypes, and (6) combined multiple measurements per cell [5].

**Fig. 2:**
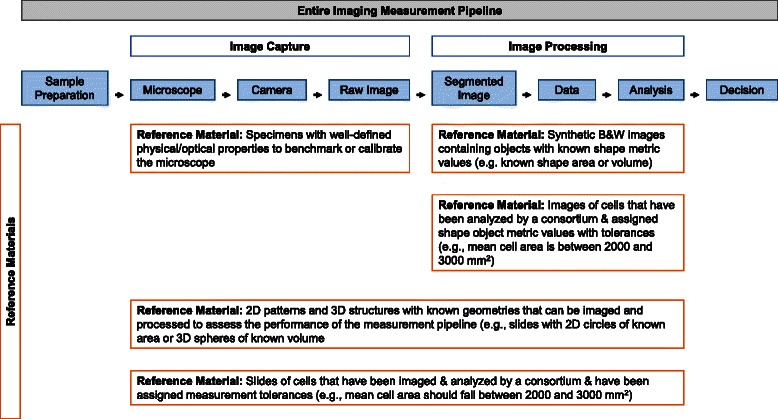
Top: The pipeline for an imaging measurement. Bottom: Different types of reference materials that can be used to evaluate performance of the different stages of the measurement pipeline

These cellular measurements from segmentation results depend on the entire imaging measurement pipeline shown in Fig. 2.

The pipeline for an imaging measurement is broken down into three stages: sample preparation, image capture and image processing. Reference materials, organized by constituent parts (Fig. 2, orange boxes), can be used to evaluate the performance of the stages of the pipeline.

### Survey usefulness and organization

This survey paper reports statistics of classification categories for automated segmentation methods. The segmentation classification categories are introduced to provide multiple perspectives on an image segmentation step. Segmentation can be viewed from the perspective of a cell biologist as a cellular measurement, or from the perspective of a computer scientist as an algorithm. Both, cell biologists and computer scientists, are interested in analyzing accuracy, error, and execution speed of segmentation (i.e., evaluation perspective of segmentation) as applied to cell measurements. We establish multiple categories for various perspectives on segmentation and classify each paper accordingly.

The term “statistics” refers to frequencies of occurrence and co-occurrence for the introduced classification categories. The occurrence and co-occurrence values are also known as 1^st^ and 2^nd^ order statistics. The terms “survey statistics” indicate that we perform a survey of papers, classify them into categories, and then report statistics of the categories.

The usefulness of survey statistics lies in gaining the insights about the community-wide usage of segmentation. With this insight, a principal investigator who is not interested in applying segmentation to his images can classify his/her cellular measurement problem and follow the most frequently used segmentation in the community. Thus, his work focusing on other aspects of cell biology can just refer to all other papers that have been reported with the same segmentation method. He can justify the segmentation choice based on the usage statistics in the cell biology community. On the other hand, a principal investigator who is interested in doing segmentation research can gain insights about which segmentation methods have not been applied to certain cellular measurements and hence explore those new segmentation approaches.

Overall, this surveys aims at understanding the state-of-the-art of cellular measurements in the context of the imaging measurement pipeline yielding segmented objects. Following from Fig. 2 cellular measurements have an intrinsic accuracy, precision, and execution speed that depend on steps of the pipeline. In order to understand the attributes of cellular measurements, we performed a survey of published literature with the methodology described in Methods section. The segmentation-centric steps of the imaging measurement pipeline are outlined in Results section. Statistical summaries of classified publications can be found in Discussion section. Finally, Conclusions section presents a list of open research questions based on our observations of the published papers.

## Methods

This survey was prepared based on an iterative process denoted in literature as “a cognitive approach” [6]. This approach starts with an initial definition of the scope of this survey (i.e., see the search filters in Endnotes section) and a classification schema. All found and manually filtered publications are classified into the categories presented in Table 1. For the purpose of this survey, the classification includes main categories of (1) objects of interests (or objects to be segmented), (2) imaging modalities, (3) digital data axes, (4) segmentation algorithms, (5) segmentation evaluations, (6) computational hardware platforms used for segmentation acceleration, and (7) object (cellular) measurements. The sub-categories in Table 1 come from specific taxonomies that are introduced in the sub-sections of Results section.

**Table 1:** Seven main classification criteria of publications (columns) and their categories

Object of interest	Imaging modality	Data axes	Segmentation	Segmentation evaluation	Segmentation acceleration	Object^a^measurement
Cell	Phase contrast	X-Y-T	Active contours + Level Set	Visual inspection	Cluster	Geometry
Nucleus	Differential interference contrast	X-Y-Z	Graph-based	Object-level evaluation	Graphics Processing Unit (GPU)	Motility
Synthetic (digital model)	Bright-field	X-Y-Z-T	Morphological	Pixel-level evaluation	Multi-core CPU	Counting
Synthetic (reference material)	Dark-field		Other	Technique is not specified	Single-core Central Processing Unit (CPU)	Location
Other	Confocal fluorescence		Partial Derivative Equations	Unknown	Unknown	Intensity
	Wide-field fluorescence		Region growing			
	Two-photon fluorescence		Thresholding			
	Light sheet		Watershed			

^a^Object refers to the categories of an object of interest and clusters of objects

The categories of objects of interest were chosen based on foci of cell biology studies and capabilities of optical microscopy. We have selected cell, nucleus, and synthetically generated objects generated using a digital model or a reference material. Synthetic objects are used for segmentation evaluations. The category “Other” includes, for instance, Golgi apparatus boundary, extracellular space, heterochromatin foci, olfactory glomeruli, or laminin protein.

The segmentation categories are based on published techniques across a variety of applications domain. They follow standard categories (e.g., thresholding, region growing, active contours and level set) in segmentation surveys [7–9] with additional refinements (e.g., watershed, cluster-based, morphological, or Partial Derivative Equations (PDEs)). The taxonomy for segmentation categories is presented in Design of automated segmentation algorithms section.

The evaluation of automated segmentation is categorized according to the level of automation as visual inspection (i.e., manual) and object-level or pixel-level evaluation. The object-level evaluation is concerned with the accuracy of the number of objects and/or approximate location, for example, in the case of counting or tracking. The pixel-level evaluation is about assessing accuracy of object shape and location, for instance, in the case of geometry or precise motility. Some papers do not report evaluation at all (classified as “unknown”) while others report results without specifying a segmentation evaluation method (classified as “technique is not specified”).

The categories of segmentation acceleration reflect current computational hardware platforms available to researchers in microscopy labs and in high-throughput biological environments. The platforms include single-core CPU (central processing unit), multi-core CPU, GPU (graphics processing unit), and computer cluster. We have not found a segmentation paper utilizing a supercomputer with a large shared memory. In addition, some researchers report a multi-core CPU hardware platform but do not mention whether the software was taking advantage of multiple cores (i.e., algorithm implementations are different for multi-core CPU than for single-core CPU platforms). Papers that do not report anything about a computational platform or the efficiency of segmentation execution are placed into the category “Unknown”.

Finally, the object or cellular measurement categories are derived from five types of analyses that are performed with 2D + time or 3D cell imaging. These analyses are related to motility, shape, location, counting, and image intensity. They are the primary taxa for mammalian cell image segmentation. Any other specific types of analyses were included in these main five classes or their combinations. For instance, monitoring cell proliferation would be classified as motility and counting or abundance quantification of intracellular components would be classified as location and counting.

While we went over close to 1000 publications and cross-referenced more than 160 papers, we classified only 72 papers according to the above criteria. We excluded from the classification publications that presented surveys or foundational material, did not include enough information about a segmentation method, or were published before the year 2000. Co-authors of this survey sometimes included a few of these papers into the main text to refer to previously published surveys, to seminal publications, or to the key aspects of segmentations demonstrated outside of the scope of this survey. Thus, there is a discrepancy between the number of classified and cross-reference papers. The 72 papers went through independent classifications by at least two co-authors. If a different category was assigned by two co-authors then a third co-author performed another independent classification. Although this verification process doubled the amount of work, we opted for classification quality rather than for quantity given our limited resources.

Our method for validating the classification schema presented above is to compute the occurrence of papers that fall into each category, and the co-occurrence of the classification categories in each paper. The list of papers that are contributing to each occurrence or co-occurrence number are converted programmatically into a set of hyperlinked web pages and can be browsed through at https://isg.nist.gov/deepzoomweb/resources/survey/index.html. The publications and their statistical summaries can be interpreted not only for validation purposes (low values suggest removing a segmentation category from classification) but also for identifying segmentation methods that have not been applied to optical microscopy images of mammalian cells.

## Results

**Fig. 3:**
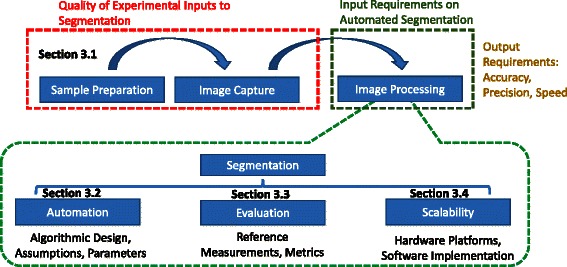
Survey organization of the Results section with respect to the imaging measurement pipeline. Four sections are devoted quality of segmentation inputs (Experimental inputs to cell imaging and segmentation), automation (Design of automated segmentation algorithms), evaluation (Evaluations of automated segmentations) and computational scalability (Scalability of automated segmentations)

We organized the results into four main sub-sections devoted to (1) experimental inputs to segmentation, (2) automated segmentation, (3) evaluation of automated segmentation, and (4) hardware platforms for computational scalability of automated segmentation as illustrated Fig. 3. The sections have a direct relationship to the imaging pipeline presented in Fig. 2.

Due to the typical variations in microscopy image appearance, it is important to understand experimental cell imaging inputs to automated segmentation. Variations in cells, reagents, and microscope instrumentations have a great impact on segmentation accuracy [10]. Thus, the design of an automated segmentation algorithm is driven by sensitivity of segmentation to the variations in cell imaging inputs.

The choice of automated segmentation technique can be facilitated by our understanding of segmentation algorithm design, particularly the assumptions for image invariance, the mathematical model for obtaining segments, and the model parameters. Numerical representations of a mathematical model and techniques for optimizing model parameters can also vary across implementations of the same automated segmentation method and determine performance robustness to extreme inputs.

Evaluations of automated segmentation are critical for the comparison-based choice of a segmentation algorithm, for optimization of segmentation parameters, and for the dynamic monitoring of segmentation results to guarantee performance and consistency. However, evaluations depend on designing task-specific metrics and on either reference segmentation for supervised evaluations or an objective cost function for unsupervised evaluations.

Finally, with the continuous advancements in microscopy, automated segmentations are deployed in increasingly diverse research and industrial settings and applied to exponentially growing volumes of microscopy images. In order to create cost effective solutions when segmenting large amounts of images, computational scalability of segmentation on a variety of hardware platforms becomes a selection criterion and has to be included in the evaluations. With the emphasis on reproducibility of biological experiments, computational scalability is not only of interest to bio-manufacturing production environments but also to research institutions conducting large scale microscopy experiments to achieve high statistical confidence of findings.

### Experimental inputs to cell imaging and segmentation

While optical microscopy is frequently used as a qualitative tool for descriptive evaluations of cells, the tool is used increasingly to generate digital images that are segmented and used to measure the shape, arrangement, location and the abundance of cellular structures or molecules. There are many advantages to quantitative analysis by automated segmentation algorithms including the capability to assess large datasets generated by automated microscopy in an unbiased manner. In the absence of computational analysis, researchers are often limited to comparatively small sample sizes and presenting microscopic data with a few “look what I saw” images.

The cellular measurements derived from image segmentation can be strongly influenced by specimen preparation [11] and the instrumentation [12] used to image the specimens. The single most important factor for good segmentations is high contrast between foreground and background, and this is achieved by carefully considering four inputs: (1) Cells, (2) Reagents, (3) Culture Substrate/Vessels, and (4) Optical Microscopy Instrumentation. Common sources of variability from these inputs are outlined in Table 2 and should be carefully managed in order to provide high foreground intensity and low background intensity. Images from the initial observations that characterize a new biological finding are not always the best for quantitative analysis. Refinement and optimization of the sample preparation and the imaging conditions can often facilitate quantitative analysis. In the overview of the four experimental inputs, we highlight reports that have used experimental techniques to improve or facilitate downstream segmentation and analysis. Interested readers can consult in-depth technical reviews and books on reagents [13–16], culture substrate/vessels [17–19], and optical microscopy instrumentation [20–22], The single most important factor for good segmentations is high contrast between foreground and background, and this is achieved by carefully considering four Common sources of variability from these inputs are outlined in Table 2 and should be carefully managed in order to provide high foreground intensity and low background intensity [1].

**Table 2:** Sources of variability in a quantitative optical microscopy pipeline and methods for monitoring and assuring data quality

Stage of pipeline	Measurement assurance strategy	Source of variability assessed/addressed	Reference
Sample Preparation	-Establish well-defined protocols for handling cells (ASTM F2998)	Cell culture variability (cell type, donor, passage, history, culturing protocol, user technique)	[23, 94]
	-Use stable and validated stains (e.g. photostable, chemically stable, high affinity, well characterized antibody reagents)	Instability of probe molecule and non-specific staining	[95–98]
	-Choose substrate with low and homogeneous background signal for selected imaging mode or probe (ASTM F2998)	Interference from background	[94, 99–101]
	-Optimize medium [filter solutions to reduce particulates, reduce autofluorescence (phenol red, riboflavin, glutaraldehyde, avoid proteins/serum during imaging)		
	-Optimize experimental design to the measurement (e.g., low seeding density if images of single cells are best) (ASTM F2998)	Interference from cells in contact	[94, 102]
Image Capture	-Use optical filters to assess limit of detection, saturation and linear dynamic range of image capture (ASTM F2998)	Instrument performance variability (e.g.) light source intensity fluctuations, camera performance, degradation of optical components, changes in focus)	[94, 103, 104]
	-Optimize match of dyes, excitation/emission wavelength, optical filters & optical filters	Poor signal and noisy background	[105, 106]
	-Minimize refractive index mismatch of objective, medium, coverslips & slides		
	-Use highest resolution image capture that is practical (e.g., balance throughput with magnification, balance numerical aperture with desired image depth)		
	-Calibrate pixel area to spatial area with a micrometer	Changes in magnification	[107, 108]
	-Collect flat-field image to correct for illumination inhomogeneity (ASTM F2998)	Non-uniformity of intensity across the microscope field of view	[94, 109–112]

A final, but critical aspect of the inputs for cell imaging experiments is documenting metadata about how cells, reagents and instrumentation were used [23]. Storing and accessing metadata describing a cellular imaging experiment has been the focus of several research efforts including, ProtocolNavigator [24] and the Open Microscopy Environment project [25, 26]. This metadata serves as evidence for measurement reproducibility in the cell image experiments. The irreproducibility of biological studies has recently be highlighted [27, 28]. A benefit to performing cellular studies using measurements derived from image segmentation is that they can, in principle, be quantitatively reproduced. This means that statistics can be applied to the data to determine the measurement uncertainty. Because the measurement uncertainty depends on the experimental inputs, methods that can be used to monitor each input can be valuable for assuring the reproducibility of a complex, quantitative imaging pipeline. A tabulated list of sources of variability and reference protocols and materials that can be used to monitor the measurement quality in a quantitative cell imaging analysis pipeline prior to segmentation are provided in Table 2.

### Input: cells

We focus this survey on the imaging of cultured mammalian cells because of the critical role these systems play in drug screening, medical diagnostics, therapies, and basic cell biology research. The complexity of cellular features observed during imaging can lead to challenging segmentation problems. At the population level, mammalian cells exhibit substantial phenotypic heterogeneity [29], even among a genetically homogeneous population of cells. This means that it is important to image and measure large numbers of cells in order to obtain statistical confidence about the distribution of phenotypes in the population.

Despite the challenges associated with segmenting and identifying cells, in some cases experimental approaches can be selected to facilitate automated analysis and segmentation. In a recent example of this used by Singer et al. [30], a histone-GFP fusion protein was placed downstream of the Nanog promoter in mouse pluripotent stem cells. In this way, the Nanog reporter was localized to the nucleus. A similar example was used by Sigal et al. to probe the dynamic fluctuations exhibited by 20 nuclear proteins [31]. Without nuclear localization, the image analysis would have been substantially more challenging as cells were frequently touching, and the boundary between cells was not well defined in the images. In such cases, a few considerations in the design of the cellular specimen to be imaged can greatly reduce the complexity of algorithms required for segmentation and improve the confidence in the numerical results.

### Input: reagents

Reagents used as indicators for cellular function or as labels for specific structures are central to quantitative imaging experiments. The development of probes has a rich history and researchers have access to a large number of probe molecules, including labeled antibodies, through commercial vendors. A description of two recent surveys of probes is provided below so that interested readers can navigate the wide range of technologies that are available. Giuliano et al. produced a particularly relevant review of reagents used within the context of high content imaging [16]. Their work provides a very good overview of the types of probes used in fluorescence microscopy and how they can be applied as physiological indicators, immunereagents, fluorescent analogs of macromolecules, positional biosensors, and fluorescent protein biosensors. In evaluating a fluorescent reagent, they consider the following six critical probe properties: fluorescence brightness (resulting from high absorbance and quantum efficiency), photostability, chemical stability, phototoxicity, non-specific binding, and perturbation of the reaction to be analyzed. Many references to the papers describing the original development of the probes themselves are included in the survey. Another relevant review was produced by the authors Kilgore, Dolman and Davidson who survey reagents for labeling vesicular structures [13], organelles [14], and cytoskeletal components [15]. This work includes experimental protocols as well as citations to original articles where the probes were applied.

### Input: culture substrate/vessel

Cells are cultured on many different types of surfaces. From the perspective of collecting digital images prior to quantitative analysis, the ideal tissue culture substrate would be completely transparent at all relevant wavelengths, non-fluorescent, defect free and have a spatially flat surface. These features would facilitate segmentation because the substrate/vessel would not produce any interfering signal with the structures of interest in the image. In practice, cells are frequently cultured on tissue culture polystyrene (TCPS) or glass, both of which are suitable for subsequent analysis particularly at low magnification.

A confounding factor for analysis of digital images of cells is that substrates are frequently coated with extracellular matrix (ECM) proteins that are necessary for the proper growth and function of the cells. The protein coating can make segmentation more challenging by adding texture to the background, both by interfering with transmitted light or by binding probe molecules thus becoming a source of background signal that can interfere with accurate segmentation [32]. Using soft lithography to place ECM proteins in on a surface in a 2-dimensional pattern can simplify segmentation by confining cells to specific locations and shapes. This approach facilitated the quantification of rates of fluorescent protein degradation within individual cells [33]. The approach of patterning has also been used to facilitate live cell analysis of stem cell differentiation. Ravin et al. used small squares patterned with adhesive proteins to limit the migration of neuronal progenitor cells to a field of view and that allowed for lineage progression within these cells to be followed for multiple generations [34]. Without patterning the image analysis problem is challenging because it requires both accurate segmentation from phase contrast or fluorescent images and tracking of cells as they migrate.

### Input: optical microscopy instrumentation

The particular image acquisition settings for imaging cells will have a profound impact on the segmentation results, as has been shown by Dima et al. [10]. Therefore, selecting the appropriate instrumentation and optimal acquisition settings is critical. General guidelines for choosing appropriate instrumentation are provided in Frigault et al. in a flow chart [22]. The authors of this article focus on live cell imaging in 3D, but the flow chart can be applied to a wide range of cell imaging experiments. The choice of instrumentation will depend on the cellular specimen, the reagents used and the substrate. When it comes to selection of the imaging mode, the goals of the qualitative visualization and quantitative analysis are the same: to image the objects under conditions that optimize the signal-to-noise ratio with minimal sample degradation. Therefore, the decision for how to image the biological sample is the same for visualization and quantitative analysis.

While it can be argued that 3 dimensional culture of cells is more physiologically relevant than culturing cells on 2 dimensional substrates [35], imaging cells on 3D scaffolds is more difficult. Cells on scaffolds are often imaged using optical sectioning techniques (i.e., confocal) to reduce the large amount of out-of-focus light that can obscure image details.

For confocal imaging, chromatic aberrations are increased along the Z-axis causing the Z-resolution to be approximately 3 times worse than the X-Y plane [36, 37]. This causes blurring in the Z-direction where spheres appear as ellipsoids. Deconvolution algorithms have been used to remove blurring, but they can be difficult to implement since they are highly dependent on imaging parameters: excitation/emission wavelengths, numerical aperture and refractive indices (RI) of the sample, medium, optics and scaffolds. A panel of reference spheres with narrow diameter distributions (15 μm +/− 0.2 μm) that are labelled with a variety of fluorescent dyes [37] can be used to assess the Z-axis aberrations for different wavelength fluorophores, but the reference spheres are not perfect mimics for cells due to differences in RI. References spheres are made of polystyrene, RI of 1.58; RI of phosphate buffered saline is 1.33; RI of culture medium is 1.35; and the RI of cells is challenging to measure, may depend on cell type and has been observed to be within the range of 1.38 to 1.40 [36, 38, 39].

In addition, the scaffolds used for 3D culture interfere with imaging. Non-hydrogel forming polymers, such as poly(caprolactone), can block light and obscure portions of cells that are beneath scaffold struts. Hydrogel scaffolds, such as cross-linked poly(ethylene glycol) (PEG), collagen, fibrin or matrigel scaffolds, can have differing refractive indices causing chromatic aberrations and light scattering effects in the imaging. In addition, hydrogel samples can have spatial inhomogeneities (polymer-rich or -poor phases) that can blur light. Some flat materials may be reflective and bounce light back into the detector resulting in imaging artifacts.

A potential solution could be the development of reference spheres with RIs that match cells. These could be spiked into cells during seeding into 3D scaffolds, and then beads could be imaged along with the cells. In this way, the reference spheres would be imaged under conditions identical to the cells, which would allow calibration of cell measurements against the reference spheres. A potential candidate could be PEG-hydrogel spheres containing fluorophores. Fabricating highly spherical PEG spheres with a narrow diameter distribution may be a challenge. Multi-photon absorption photopolymerization can generate highly uniform structures at 10 μm size scales and may be capable of achieving this goal [40].

### Design of automated segmentation algorithms

Here, we focus on the design of segmentation methods encountered in cellular and subcellular image processing with two dimensional time sequence (x, y, t), three dimensional (x, y, z) or three dimensional time sequence (x, y, z, t) datasets. These image datasets are acquired using a subset of optical microscopy imaging modalities, such as phase contrast, differential interference contrast (DIC), confocal laser scanning, fluorescent, and bright/dark field.

Next, we describe common segmentation algorithms, their assumptions, models, and model parameters, as well as the parameter optimization approaches. In comparison to previous surveys about cell microscopy image segmentation [7], we provide more detailed insights into the design assumptions and parameter optimization of segmentation methods.

### Algorithmic design and assumptions

**Fig. 4:**
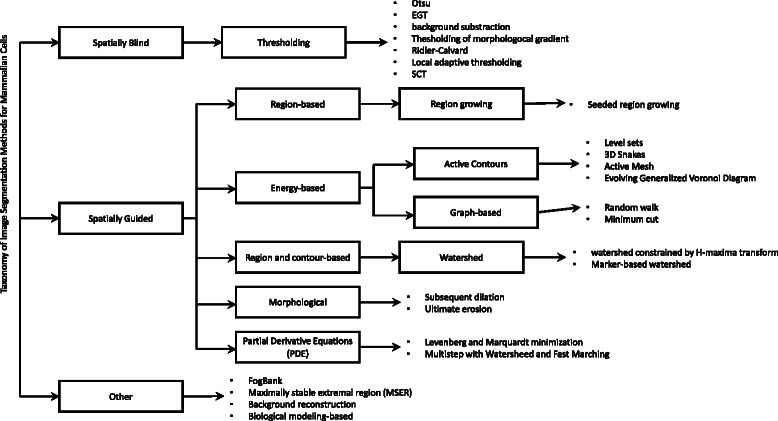
Taxonomy of image segmentation methods for mammalian cells

We classified each paper found in the literature into eight segmentation categories. The categories for our classification are derived from a general taxonomy presented in [41]. Figure 4 shows the used taxonomy of image segmentations for mammalian cells. Table 3 shows eight categories and the frequency of papers using a segmentation method from each category. The categories are used in a disproportionate manner. Threshold based techniques are the simplest and most commonly used techniques in the literature, followed by Active contours. The third most common category is Watershed and the fourth category is the custom made segmentations. In our work, if a paper described a method with multiple different approaches, like thresholding followed by watershed then this paper was classified in both thresholding and watershed categories. Co-occurrence of cellular measurements and segmentation section provides more insight on segmentation methods, image modality and image dimensionality.

**Table 3:** Summary usage statistics of segmentation methods in the surveyed literature

Segmentation category	Description	Number of surveyed papers
Active contours + Level Set	Parametric curves which fit to an image object of interest. These curve fitting functions are regularized gradient edge detectors	24
Graph-based	Applies graph theories to segment regions of interest	2
Morphological	Apply morphological operations to segment or clean a pre-segmented image	2
Other	The methods in this category are created for a specific problem or cell line by a combination of existing techniques or by creating a new concept	8
Partial Derivative Equations	Groups pixels into different segment based on minimizing a cost function using partial derivatives	2
Region growing	Starts from a seed and grows the segmented regions following some pre-defined criterion	2
Thresholding	Threshold based techniques consider the foreground pixels to have intensity values higher (or lower) than a given threshold.	31
Watershed	Mainly used to separate touching cells or touching subcellular regions	15

Every segmentation technique is built with certain assumptions about input images during an algorithmic design. Segmentation assumptions affect reported accuracy of segmentation results if they are not met. Assumptions are typically specific to each segmentation algorithm and incorporate image properties of a segmented region. According to the surveyed literature about mammalian cells and segmentation, segmentation assumptions can be categorized into three classes: (1) Biological assumptions, (2) Algorithmic assumptions and (3) Image pre-processing assumptions.

Biological assumptions in a segmentation algorithm are those that are derived from the knowledge of a biologist. For example, a nucleus is considered to have a round shape or a mother cell is small, round, and bright before mitosis in phase contrast images. Algorithmic assumptions are those made during the development of a segmentation algorithm. For instance, assumptions about k-means clustered pixels as initial inputs to the level sets functions, pixel intensities being distributed according to a bi-modal distribution, etc. Image pre-processing assumptions are those that require image processing operations to be applied before segmenting the images. Examples of such pre-processing are a Hessian-based filtering or intensity binning as necessary operations for improved performance. A more detailed description about each assumption class is presented in Table 4.

**Table 4:** A summary of segmentation assumptions in the surveyed literature

Assumptions	Sub-category	Description	References
Biological assumptions	Image Contrast	Strong staining to get high SNR for actin fibers	[113]
		Optophysical principle of image formation is known	[44]
		Cell brightness significantly higher than background	[114, 115]
		Cell signal higher than noise level in an acquired z-stack	[49, 116–118]
	Object Shape	Biological assumptions about mitotic events like mother roundness and brightness before mitosis	[119–122]
		Nucleus shape is round	[123]
		Specifically designed for dendritic cells	[83]
		Cell line falls into one a few object models. Cell must have smooth borders. E.coli model assumes a straight or curved rod shape with a minimum volume darker than background. Human cells assume nearly convex shape.	[124]
		Cells posses only one nucleus	[125]
Algorithmic assumptions	Background/Foreground Boundary	Initializing level sets functions based on k-means clustering	[126]
	Background	Background intensities are between the low and high intensities in the image	[127]
		Local background must be uniform	[128, 129]
		Background is piecewise linear and its intensities are between the low and high intensities in the image	[130]
	Foreground	Clear distinction between touching cell edge pixel intensities	[122]
		Foreground pixels are drawn from a different statistical model than the background pixels	[131]
		Features computed based on their gray-scale invariants	[132]
	Time	The first image of a time sequence should be segmented first by another algorithm like watershed	[69]
	Intensity Distributions	Image pixel intensities follow bi-modal histogram	[42]
		The statistical properties of the foreground and background are distinct and relatively uniform & foreground is bright, while the background is dark	[133]
		Foreground and background follow Gaussinan distribution	[134]
Image pre-processing	Background flatfield correction	Image pre-processing: such as correcting inhomogeneous illuminated background intensities using a machine learning based approach to resolve differences in illumination across different locations on the cell culture plate and over time	[81]
	Filters	Smoothing the image using Gaussian filter	[132]
		Downsampling (binning) the images	[64]
		Image smoothing and automatic seed placement are used	[56]
		Hessian-based filtering for better cell location and shape detection	[44]
	Non-linear transformation	Image pre-conditioning where the image is transformed to bright field before applying the threshold	[48]
	Manual input	Manual interactivity is needed to compute segmentation	[84]

### Tools, packages, code availability, languages

Several software packages include segmentation algorithms that can be applied to images across different imaging modalities and cell lines. These packages range from polished tools with graphical interfaces to simple collections of segmentation libraries. A recent survey of biological imaging software tools can be found in [26]. The list provided in Table 5 includes tools with segmentation software packages encountered during this literature survey of segmentation techniques as well as methods that could potentially be used for mammalian cell segmentation. This table is inclusive to the segmentation field we are surveying but it is not by any means an exhaustive list of the available tools in that field.

**Table 5:** A summary of software packages encountered during this literature survey

Software name	Description	Tool availability	Reference
Ilastik	A tool for interactive image classification, segmentation, and analysis	S	[135]
FARSIGHT	Toolkit of image analysis modules with standardized interfaces	S	[136]
ITK	Suite of image analysis tools	S	[137]
VTK	Suite of image processing and visualization tools	S	[138]
CellSegmentation3D	Command line segmentation tool	E	[139]
ImageJ/Fiji	Image processing software package consisting of a distribution of ImageJ with a number of useful plugins	E + S	[78]
Vaa3D	Cell visualization and analysis software package	E + S	[140]
CellSegM	Cell segmentation tool written in MATLAB	S	[141]
Free-D	Software package for the reconstruction of 3D models from stacks of images	E	[142]
CellExplorer	Software package to process and analyze 3D confocal image stacks of C. elegans	S	[143]
CellProfiler	Software package for quantitative segmentation and analysis of cells	E + S	[144]
Kaynig’s tool	Fully automatic stitching and distortion correction of transmission electron microscope images	E + S	[145]
KNIME	Integrating image processing and advanced analytics	E + S	[146]
LEVER	Open-source tool for segmentation and tracking of cells in 2D and 3D	S	[31, 147]
OMERO	Client–server software for visualization, management and analysis of biological microscope images.	E + S	[148]
Micro-Manager	Open-source microscope control software	E + S	[149]
MetaMorph	Microscopy automation and image analysis software	PE	[124]
Imaris	Software for data visualization, analysis, segmentation, and interpretation of 3D and 4D microscopy datasets.	PE	[150]
Amira	Software for 3D and 4D data processing, analysis, and visualization	PE	[151]
Acapella	High content imaging and analysis software	PE	[85]
CellTracer	Cell segmentation tool written in MATLAB	E + S	[124]
FogBank	Single cell segmentation tool written in MATLAB	E + S	[122]
ICY	Open community platform for bioimage informatics.	E + S	[65]
CellCognition	Computational framework dedicated to the automatic analysis of live cell imaging data in the context of High-Content Screening (HCS)	E + S	[152]

Tool Availability options are (P)roprietary, (E)xecutable Available, (S)ource Available

### Optimization of segmentation parameters

In many cases, segmentation techniques rely on optimizing a parameter, a function or a model denoted as optimized entities. The goal of optimizing these entities is to improve segmentation performance in the presence of noise or to improve robustness to other cell imaging variables.

Around 40 % of the surveyed papers do not mention any specific parameter, function or model optimization. Based on the remaining 60 % of the papers, five categories of optimization entities were identified: (1) intensity threshold, (2) geometric characteristics of segmented objects, (3) intensity distribution, (4) models of segmented borders, and (5) functions of customized energy or entropy. Almost 50 % of the papers that explicitly mention parameter optimization rely on intensity threshold and/or intensity distribution optimization. Parameters related to the segmented object geometry are optimized in approximately 30 % of the papers while models of segmented border location are optimized in approximately 15 % of the surveyed publications. The remaining 5 % describe algorithms that make use of customized energy or entropy functions, whose optimization leads to efficient segmentation for specific applications.

Table 6 illustrates a summary of five representative publications for the most highly occurring categories of optimization entities (categories 1, 2 and 3 above) in terms of the use of optimization.

**Table 6:** A summary of five publications in terms of their use of segmentation parameter optimization

Optimized entity	Optimization approach	Segmentation workflow	Reference
Intensity threshold, intensity distribution	Otsu technique [43] to minimize intra-class variance	Thresholding→Morphological seeded watershed	[42]
		DIC-based nonnegative-constrained convex objective function minimization→ Thresholding	[44]
Intensity threshold, intensity distribution, geometric characteristics of segmented objects	Find threshold that yields expected size and geometric characteristics	Gaussian filtering→Exponential fit to intensity histogram→Thresholding→ Morphological refinements	[49]
		Thresholding→Morphological refinements	[47]
Intensity distribution, geometric characteristics of segmented objects	Hessian-based filtering and medial axis transform for enhanced intensity-based centroid detection	Iterative non-uniformity correction→Hessian-based filtering→Weighted medial axis transform→Intensity-based centroid detection	[48]

Table 6 also shows how the segmentation workflow often consists of a number of steps, such as seeded watershed, various image filters, medial axis transform, and morphological operations, which involve different optimization entities. For instance, Al-Kofahi et al. [42] employ Otsu thresholding [43], followed by seeded watershed in order to correctly segment large cells. Bise et al. [44] eliminate differential interference contrast (DIC) microscopy artifacts by minimizing a nonnegative-constrained convex objective function based on DIC principles [45], and then the resulting images are easily segmented using Otsu thresholding [43]. Ewers et al. [46] initially correct for background and de-noise using Gaussian filters. Local intensity maxima are then sought based on the upper percentile, and are optimized based on the (local) brightness-weighted centroid and on intensity moments of order zero and two. We found several examples of intensity thresholding combined with geometry-based refinements [47], iterative procedures [48], and global fitting steps [49].

Interesting optimization approaches can be also found in applications of segmentation methods outside of the scope of this survey. Such segmentation methods use for instance artificial neural networks (ANN) and optimize ANN weights [50], 3D active shape models (ASM) and optimize shape variance [51], or geometrically deformable models (GDM) which rely on finding optimal internal and external forces being applied to deform 2D contours [52].

### Evaluations of automated segmentations

We focus on accuracy and precision evaluations of automated segmentation algorithms. The evaluation approaches have been classified according to the taxonomy in [53]. They have been expanded by object and pixel level evaluations in Table 7. The object level evaluation is important for the studies focusing on counting, localization or tracking. The pixel level evaluation is chosen for the studies measuring object boundaries and shapes.

**Table 7:** Taxonomy of segmentation evaluation approaches

Taxonomy of segmentation evaluation	Subjective				
	Objective	System Level			
		Direct	Analytical		
			Empirical	Unsupervised	Object level (counts, centroids)
					Pixel level (boundaries)
				Supervised	Object level (counts, centroids)
					Pixel level (boundaries)

The majority of evaluations found in the literature of interest to this survey fall under empirical methods with supervised and unsupervised evaluation approaches.

Next, we overview both unsupervised and supervised segmentation evaluation approaches and highlight several segmentation quality criteria and metrics, as well as challenges with creating reference segmentation results and selecting samples for the reference segmentations. Finally, we summarize evaluations methods employed in several past grand segmentation challenges that have been conducted in conjunction with bio-imaging conferences.

### Unsupervised empirical evaluation design

Unsupervised evaluation of segmentation methods are also known as stand-alone evaluation methods and empirical goodness methods. A relatively broad survey of such methods is presented in [53]. Unsupervised evaluations do not require a creation of ground truth segmentation. Thus, they scale well with the increasing number of segmentation results that have to be evaluated for accuracy. Furthermore, these methods can be used for tuning segmentation parameters, detecting images containing segments with low quality, and switching segmentation methods on the fly.

In this class of evaluation methods, the goodness of segmentation is measured by using empirical quality scores that are statistically described, and derived solely from the original image and its segmentation result. One example of a quality score is the maximization of an inter-region variance in threshold-based Otsu segmentation [43]. Unfortunately, there is no standard for unsupervised evaluation of automated segmentation because the segmentation goodness criteria are application dependent. Moreover, application and task specific criteria are often hard to capture in a quantitative way because they come from descriptions based on visual inspections. As a consequence, unsupervised segmentation evaluations are rarely reported in the literature focusing on optical 2D and 3D microscopy images of cells. We did not find a single paper that reported comparisons of task-specific segmentation methods using unsupervised evaluation methods. On the other hand, a few researchers utilized elements of unsupervised evaluations in their segmentation pipeline in order to improve their final segmentation result. We describe three such examples next.

Lin et al. in [54] and [55] segment cellular nuclei of different cell types. The initial segmentation is performed with a modified watershed algorithm to assist with nucleus clustering and leads to over-segmentation. The authors estimate the confidence in segmenting a nucleus as the object composed of a set of connected segments with a probability. This probability can be seen as an unsupervised segmentation quality score and is used for merging of connected segment into a nucleus object.

Padfield et al. in [56] perform a segmentation of a spatio-temporal volume of live cells. The segmentation is based on the wavelet transform. It results in the 3D set of segmented “tubes” corresponding to cells moving through time. Some of the tubes touch at certain time points. The authors use the likelihood of a segment being a cell-like object as an unsupervised segmentation score for merging or splitting separate cell tracks.

Krzic et al. in [57] segment cellular nuclei in the early embryo. The initial segmentation is performed by means of local thresholding. The authors use volume of the candidate object as a score for the decision whether the volume split operation should be performed. If the volume is greater than the mean volume plus one standard deviation then the watershed algorithm is applied to the candidate object.

### Supervised empirical evaluation design

Supervised empirical evaluation methods, also named empirical discrepancy methods are used to evaluate segmentations by comparing the segmented image against a ground-truth (or gold-standard) reference image. These methods often give a good estimation of the segmentation quality, but can be time-consuming and difficult for the expert in charge of manually segmenting the reference images. We overview publications that address a few challenges related to the creation of a reference segmentation, sampling, and evaluation metrics.

#### Creation of databases with reference cell segmentations

There is growing availability of reference segmentations on which to evaluate segmentation methods. A number of research groups have created databases of images and segmentation results that span a range of imaging modalities, object scales, and cellular objects of interest. Reference images are needed to test 3D segmentation algorithms across the variety of imaging modalities and over a wide variety of scales from cell nuclei to thick sections of biological tissues. We summarized a few cell image databases in Table 8.

**Table 8:** Examples of reference cell image databases

Cell image databases	Biological content	Scale of objects	Axes of acquired data	References
Biosegmentation benchmark	Mammalian cell lines	Nuclear to multi-cellular	X-Y-Z	[58]
Cell Centered Database	Variety of cell lines, initial data of nervous system	Subcellular to multi-cellular	X-Y-Z, X-Y-T, X-Y-Z-T	[59]
Systems Science of Biological Dynamics (SSBD) database	Single-molecule, cell, and gene expression nuclei.	Single-molecule to cellular	X-Y-T	[153]
Mouse Retina SAGE Library	Mouse retina cells	Cellular	X-Y-Z-T	[60]

Gelasca et al. in [58] describe a dataset with images covering multiple species, many levels of imaging scale, and multiple imaging modalities, with associated manual reference data for use in segmentation algorithm comparisons and standard evaluation of algorithms. The database includes images from light microscopy, confocal microscopy, and microtubule tracking and objects from one micron to several hundred microns in diameter. They also provide analysis methods for segmentation, cell counting, and cell tracking. For each data set in the database, the number of objects of interest varies with the data set.

Martone et al. in [59] have created the Cell Centered Database for high-resolution 3D light and electron microscopy images of cells and tissues. This database offers hundreds of datasets to the public. They have developed a formal ontology for subcellular anatomy which describes cells and their components as well as interactions between cell components.

A database developed based on the work of Blackshaw et al. in [60] and accessible at http://cepko.med.harvard.edu/, contains imaging data to investigate the roles of various genes in the development of the mouse retina. Various clustering methods are available to understand the relationships between sets of genes at different stages of development. A review of biological imaging software tools summarizes the current state of public image repositories in general, including those with and without reference data sets [26], contains imaging data to investigate the roles of various genes in the development of the mouse retina.

#### Sampling of objects to create reference cell images

When creating reference cell image databases, there is a question of cell sampling. For the reference databases in Table 8, little information is available describing the sampling method and how the number of reference objects for each set is chosen, or how the variability across a population of images is found.

In general, cell image samples for inclusion into the reference database can be drawn from (1) multiple cell lines, (2) multiple biological preparations, (3) one experimental preparation with many images (X-Y-T or X–Y-Z), (4) one image containing many cells, and (5) regions of a cell. A sampling strategy would be applied to select images of cells, nuclei, or cell clusters. This topic of image sampling using fluorescence images of different biological objects has been explored by Singh et al. in [61]. By performing uniform random sampling of the acquired images and comparing their variability for different sample sizes, one can estimate the size of the image to sample to stay within a specified variance. Similarly, Peskin et al. in [62] offer a study that estimated the variability of cell image features based on unusually large reference data sets for 2D images over time. The authors showed that the range of sample numbers required depends upon the cell line, feature of interest, image exposure, and image filters.

The number of objects selected for analysis varies with the type of object in the image and its scale. Nuclear images tend to have larger numbers of objects in associated analyses. Examples include studies on rat hippocampus [55], various human tissues [63], and a variety of other species, for which the numbers of nuclei per image range from 200 to 800. These numbers are high compared with images of larger objects, such as breast cancer cells [58] or rat brain cells [55], for which the number of objects in the entire study is much lower, i.e. 50 to 150. The vast majority of studies do not explain exactly how the number of objects is selected, or the shapes of the distributions of resulting data (Gaussian or non-Gaussian).

In this survey, we encountered 44 papers that referred to different sampling methods including exhaustive sampling (13), manually selected samples (11), statistical sampling (13, random or stratified), or systematic sampling (7, regular sub-sampling of data or simply choosing the first N samples). These sampling techniques were used for selecting objects or interest to create reference segmentations. We found 49 papers that described the creation of reference segmentations by using automatic (4), semi-automatic (4), manual (38), and visual (3) approaches. The manual approaches created a reference segment representation while visual approaches provided just a high level label. There were several papers that reported creation of reference segmentations but did not report sampling of objects of interests. Some papers used combinations of sampling strategies (4) or creation methods (6).

Future research involving the availability and utility of a reference data set will depend upon the extent of efforts made to manually create sets that represent true image variability for a very wide range of applications. As more reference data is collected, one can begin to ask relevant questions about required sampling sizes for different types of applications.

#### Segmentation accuracy and precision measures

Following the classification in the survey of evaluation methods for image segmentation [9], the measures used in supervised empirical segmentation evaluation methods can be classified in four main categories: measures based on (1) the number of mis-segmented voxels, (2) the position of mis-segmented voxels, (3) the number of objects, and (4) the feature values of segmented objects. We summarized measures, metrics and cellular measurements in Table 9, and describe each category of segmentation evaluation measures next.

**Table 9:** A summary of segmentation evaluation metrics

Measures based on	Metric name	Cellular measurement	Reference
Number of Mis-segmented voxels	Jaccard	Synthetic	[65]
	Dice	Cell	[120, 129, 154]
		Synthetic	[154]
		Other	[66]
	F-Measure	Synthetic	[155]
	Adjusted Rand Index	Cell	[122]
	Custom measure	Nucleus	[61]
		Cell	[67]
	Misclassification error	Nucleus	[156]
		Other	[156]
	Accuracy (ACC)	Cell	[157, 158]
Position of mis-segmented voxels	Average distance	Cell	[56]
		Synthetic	[117]
		Other	[116]
	Root square mean of deviation	Synthetic	[159]
	Histogram of distances	Nucleus	[138]
Number of objects	Object count	Nucleus	[55, 56, 123, 160–162]
		Cell	[81, 119, 163]
	Precision/Recall	Nucleus	[54, 84]
		Cell	[44, 69, 84, 127]
	F-measure	Nucleus	[84]
		Cell	[69, 84]
	Bias index	Cell	[69]
	Sensitivity	Nucleus	[138, 164]
	Custom measure	Cell	[67]
	Cell detection rate	Cell	[165]
Feature values of segmented objects	Velocity histogram	Cell	[166]
	Object position	Nucleus	[167]
		Cell	[151, 163, 166]
		Synthetic	[168]
	Pearson’s correlation slope and intercept for velocity measurements	Cell	[166]
	Voxel intensity based	Synthetic	[159]
		Other	[73]
	Object area and shape based	Cell	[151]
		Other	[73]
	Structural index	Cell	[151]

1. Measures based on the number of mis-segmented voxels
  These measures view segmentation results as a cluster of voxels, and hence evaluate segmentation accuracy using statistics such as the Jaccard and Dice indices. These indices for a class can be written as:

1 $$ Jaccard\left(R,S\right)=\frac{\left|R{\displaystyle \cap }S\right|}{\left|R{\displaystyle \cup }S\right|} $$

2 $$ Dice\left(R,S\right)=\frac{2\left|R{\displaystyle \cap }S\right|}{\left|R\right|+\left|S\right|} $$

where R is the set of voxels of the reference segmentation and S is the set of voxels obtained by the tested algorithm. To define a metric on the entire image, one can take the average of those indices over all the classes. These two measures were the most commonly used in the reviewed papers, notably in [61, 64–68].

Another common measure is the F-measure which is based on precision and recall:

3 $$ Precision\left(R,S\right)=\frac{\left|R{\displaystyle \cap }S\right|}{\left|S\right|} $$

4 $$ Recall\left(R,S\right)=\frac{\left|R{\displaystyle \cap }\ S\right|}{\left|R\right|} $$

5 $$ F\left(R,S\right)=\frac{2* Precision\left(R,\ S\right)* Recall\left(R,\ S\right)}{Precision\left(R,\ S\right)+ Recall\left(R,S\right)} $$

where R and S have the same meaning as before. The F-measure has been used in [69, 70].

These measures based on the number of mis-segmented voxels have the advantage of being simple to compute. However, they do not take into account the location of a mis-segmented voxel. The location might be important since a mis-segmented voxel close to a segment boundary might not contribute to a segmentation error as much as one far away.

(2) Measures based on the position of mis-segmented voxels
  Measuring the segmentation discrepancy by taking into account only the number of mis-segmented voxels may not be sufficient to rank several segmentations of the same objects. While two segmentation results can be similar when measuring the number of mis-segmented voxels, they might be dissimilar when measuring positions of mis-segmented voxels. The most common measure based on positions of mis-segmented voxels is the Hausdorff distance [71]. It is defined as the maximum of the sets of minimum distances of two compared shapes and has been used to evaluate 3D nuclei segmentation in [72]. Another approach is to use the position distances between 3D boundary voxels of ground truth and segmented objects in 2D slices as used by S. Takemoto and H. Yokota in [73].

(3) Measures based on the number of objects
  Measures at voxel level have the disadvantage of measuring performance without considering aggregations of voxels that form semantically meaningful objects. Measures based on the number of objects are trying to address this issue. Depending on a specific study and its spatial resolution, the objects are usually colonies, cells or nuclei. Once semantically meaningful objects are defined, one can use the same measures as those introduced for measuring the number of mis-segmented voxels. As examples, two such studies have reported the use of the Jaccard index [74] and the F-measure [70]. With object-based measurements, however, the challenge lies in matching the objects from the automatically segmented images with the objects specified as ground truth. This step is not trivial since the automatic segmentation can result in false positives (object does not exist in ground truth), false negatives (missing object in automatic segmentation), splits (object detected as multiple objects) and merges (multiple objects detected as one object). One possible solution can be found in [74] where a reference cell R and a segmented cell S match if |*R* ∩ *S*| > 0.5 |*R*|.

(4) Measures based on the feature values of segmented objects
  Image segmentation can be viewed as a necessary step to extract properties of segmented objects. The extraction goal leads to segmentation evaluations based on one or several extracted features (properties) of a segment. The evaluation objective is to verify that features extracted from the segmented object are equivalent to features measured on the original object (reference features). In other words, conclusions derived from measured features over segmented objects will be the same for the original and the segmented object. This type of evaluation is used by S. Takemoto and H. Yokota in [73]. They use a custom similarity metric combining intensity-based and shape-based image features measurements and ranking several algorithms for a given 3D segmentation task based on the distance between feature vectors. Similarly, centroids of segments are used as features in [56] and [58] which can be viewed as an extension of measuring the position of mis-segmented voxels.

Among the aforementioned measures, the most common ones are the measures based on the number of mis-segmented voxels, such as the well-known Dice or Jaccard indices. Nonetheless, other measures can be found in literature that are based on either a custom design [61] or a combination of several existing measures [73]. It is also important to note that due to the amount of labor needed to establish 3D reference segmentation manually from volumetric data, evaluations are sometimes performed against 2D reference segmentations of 2D slices extracted from 3D volumetric data [61, 73, 75].

#### Confidence in segmentation accuracy estimates

**Fig. 5:**
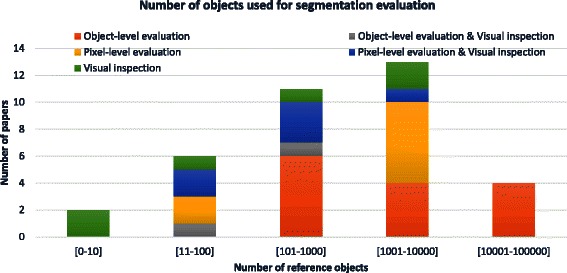
A histogram of the number of evaluation objects used in surveyed papers that reported segmentation evaluation

Sampling method and the sample size of reference objects determines the confidence in segmentation evaluation accuracy. We have extracted the information about the number of reference objects (sample size) from the classified papers and summarized them in Fig. 5. The numbers are presented per Segmentation Evaluation category introduced in Table 1. The papers that did not specify the sample size in units matching the object categories (i.e., cells, nuclei, etc.) but rather in time frames were labeled as “unknown” number of reference objects. The histogram in Fig. 5 shows 50 out of 72 papers that report the number of reference objects. It illustrates the distribution of the papers relying on qualitative/visual evaluations (2, 4, 5, 3, 0) and quantitative segmentation evaluations (0, 0, 6, 10, 4) as the number of reference objects increases.

### Evaluations of segmentation grand challenges

In the past, segmentation accuracy evaluation of biomedical images has been formulated as grand challenges by several conferences. The majority of challenges have been affiliated with the Medical Image Computing and Computer-Assisted Intervention (MICCAI) conference and the IEEE International Symposium on Biomedical Imaging (ISBI): From Nano to Macro (see http://grand-challenge.org/All_Challenges/). Other conferences, such as SPIE, ECCV, and ICPR for computer vision and pattern recognition communities, have recently introduced such biomedical image challenges as well.

Although the specific biomedical imaging domain varies in these challenges, almost all of them include a segmentation step. For example, among the grand challenges affiliated with the 2015 ISBI conference, seven out of the eight included segmentation. Out of those seven, two challenges are related to mammalian cell segmentation (Cell Tracking Challenge and Segmentation of Overlapping Cervical Cells from Multi-layer Cytology Preparation Volumes). These challenges run over two to three years since the segmentation problem remains an open problem in general. In addition, the challenges transition from segmenting 2D to 3D data sets which increases the difficulty of designing an accurate solution.

In terms of challenge evaluation, the challenge of segmentation of overlapping cervical cells is assessed using the average Dice Coefficient against manually annotated cytoplasm for each cell and nucleus, and against a database of synthetically overlapped cell images constructed from images of isolated cervical cells [76, 77]. The cell tracking challenge is evaluated using the Jaccard index, and against manually annotated objects (the ground truth) consisting of the annotation of selected frames (2D) and/or image planes (in the 3D cases) [74].

### Summary of segmentation evaluation

Evaluation of automated segmentation methods is a key step in cellular measurements based on optical microscopy imaging. Without the evaluations, cellular measurements and the biological conclusions derived from them lack error bars, and prevent others from comparing the results and reproducing the work.

The biggest challenge with segmentation evaluations is the creation of reference criteria (unsupervised approach) or reference data (supervised approach). The reference criteria are often hard to capture in a quantitative way because they are based on observations of experts’ visual inspections. As a consequence, unsupervised segmentation evaluations are rarely reported in the literature using optical microscopy images of cells. If segmentation parameters have to be optimized then some papers use “goodness criteria” for this purpose.

The challenge with creating reference data is the amount of labor, human fatigue, and reference consistency across human subjects. Software packages for creating reference segmentation results have been developed [78, 79]. These software packages provide user friendly interfaces to reduce the amount of time needed. However, they do not address the problem of sampling for reference data, and do not alleviate too much the human aspects of the creation process.

Finally, there are no guidelines for reporting segmentation evaluations. For example, evaluations of segmentation objects are summarized in terms of the total number of cells, frames or image stacks, or a sampling frame rate from an unknown video stream. These reporting variations lead to ambiguity when attempts are made to compare or reproduce published work.

### Scalability of automated segmentations

We have focused our survey of the segmentation literature on the use of desktop solutions with or without accelerated hardware (such as GPUs), and the use of distributed computing using cluster and cloud resources. These advanced hardware platforms require special considerations of computational scalability during segmentation algorithm design and execution. The categories of hardware platforms in Table 1 can be placed into a taxonomy based on the type of parallelism employed, as given in Table 10.

**Table 10:** Taxonomy of hardware platforms

Taxonomy of hardware platforms	Parallel	MIMD	Cluster
			Multi-core CPU
		SIMD	GPU
	Serial		Single-core CPU

SIMD is Single Instruction, Multiple Data streams, MIMD is Multiple Instruction, Multiple Data streams [169]

Based on our reading of the literature that meets the survey criteria, the topic of computational scalability is currently not a major concern for the 3D segmentation of cells and subcellular components. While algorithms in other application areas of 2D and 3D medical image segmentation are often developed to support scalability and efficiency [80], most of the papers we surveyed made no claims about computational efficiency or running time. Of the works that did claim speed as a feature, only a few exploited any kind of parallelism, such as computer clusters [81], GPUs [82], or multi-core CPUs [83–87]. Some other algorithms made use of the GPU for rendering (e.g. Mange et al. [83]) rather than for the segmentation itself. For algorithms that did exploit parallelism for the actual segmentation, it was generally either to achieve high throughput on a large data set (e.g. on clusters for cell detection in Buggenthin et al. [81]) or to support a real-time, interactive application (e.g. on multi-core CPUs for cell tracking in Mange et al. [83] and for cell fate prediction in Cohen et al. [88]). We did not find any works which made use of more specialized hardware, such as FPGAs.

In addition to algorithmic parallelism and computational scalability, the segmentation result representation plays an important role in the execution speed of rendering and post-processing of segments. The output of most cell segmentation algorithms is in the form of pixels or voxels, which are a set of 2D or 3D grid points, respectively, that sample the interior of the segmented region. Some other works produce output in the form of a 3D triangle mesh (e.g. Pop et al. [89]), or its 2D equivalent, a polygon (e.g. Winter et al. [87]). While the triangular mesh representation is very amenable to rendering, especially on the GPU, it is less suitable than a voxel representation for certain types of post-segmentation analysis, such as volume computation.

Spherical harmonics is another representation used in some works (e.g. Du et al. [64], Eck et al. [66]). Du et al. [64] first compute a voxel-based segmentation to which they fit spherical harmonic basis functions, while Eck et al. [66] directly compute the spherical harmonic segmentation. While in general a spherical harmonics representation takes some extra effort to render and analyze, it enables analyses such as shape comparison, as in Du et al. [64]. A disadvantage of spherical harmonics representations is that they can only represent objects with a spherical topology. Delgado-Gonzalo et al. [65] represent their segmentations as exponential B-splines, which offer fast computation and are amenable to user manipulation during semi-automatic segmentation. This representation does, however, require additional processing before rendering or analysis.

## Discussion

We summarized statistics about papers in the following ways: (1) a co-occurrence of publications that reported various types of cellular measurements and the segmentations used to obtain those measurements, (2) statistics about segmentation inputs and outputs, (3) a co-occurrence of publications that reported various types of segmented objects and evaluation approaches, and (4) statistics about segmentation software.

### Co-occurrence of cellular measurements and segmentation

Table 11 might provide insights about pairs of segmentation methods and specific cellular measurements. It could be observed that the most frequently use segmentation method is thresholding with a variety of threshold optimization approaches (see a survey devoted to the optimization topic in [90]. The papers classified under “Other” segmentation methods are many times using thresholding but are a part of a multi-stage complex algorithm. The hyperlinks in the table point to the web pages with the list of publications.

**Table 11:** Co-occurrence Statistics of Surveyed Publications: Segmentation Method versus Cellular Measurements

	Thresholding	Watershed	Region growing	Active contours + Level Set	Other	Morphological	Graph-based	Partial derivative equations (PDE)
Motility	11	4	1	12	2	1	0	0
Counting	6	4	1	1	4	0	0	0
Location	8	7	0	7	2	1	1	1
Geometry	9	1	1	6	3	0	2	1
Intensity	3	3	0	0	0	0	0	0

Tables 12 and 13 offer similar statistics but with regards to Imaging Modality and data Dimensionality. These three tables are very useful as a guide to what was used to segment images similar to one’s experiment. For example from Table 11, one can conclude that watershed was not used to measure cell geometry but rather active contours and threshold were mainly used for that measurement. From Table 12 a developer of segmentation algorithm may consider to use a custom built segmentation method for segmenting objects in DIC image modalities since none of the popular segmentation methods were used on that imaging modality. These three tables should be a start to narrow down the research papers and the segmentation methods used to solve a similar project at hand.

**Table 12:** Co-occurrence Statistics of Surveyed Publications: Segmentation Method versus Imaging Modality

	Phase contrast	Wide-field fluorescence	Bright-field	Confocal fluorescence	Differential interference contrast	Dark-field	Two-photon fluorescence	Light sheet
Thresholding	10	11	2	11	3	1	1	0
Watershed	4	11	1	5	0	0	0	1
Region growing	0	1	1	0	0	0	0	0
Active contours + Level Set	3	5	2	15	0	0	0	0
Other	5	2	3	2	1	0	0	0
Graph-based	0	1	0	1	0	0	0	0
Partial Derivative Equations (PDE)	0	0	0	2	0	0	0	0
Morphological	0	0	0	1	1	0	0	0

**Table 13:** Co-occurrence Statistics of Surveyed Publications: Segmentation Method versus Axes of Digital Data

	X-Y-T	X-Y-Z	X-Y-Z-T
Thresholding	19	13	2
Watershed	9	5	1
Region growing	2	0	0
Active contours + Level Set	11	9	4
Other	7	2	0
Graph-based	1	0	1
Partial Derivative Equations (PDE)	0	1	1
Morphological	1	1	0

### Statistics about inputs and outputs

The reviewed publications reported 40 data sets with X-Y-T dimensions, 27 data sets with X-Y-Z dimensions, and 7 data sets with X-Y-Z-T dimensions. Of the works that used X-Y-T, most treated T as a separate dimension, first performing an algorithm on each X-Y slice separately and then iterating over T. However, some works (e.g. Padfield et al. [56, 91]) treated X-Y-T as a unified 3D volume and performed an algorithm over all dimensions simultaneously.

In terms of imaging modalities Table 14 shows that confocal fluorescence imaging is the most frequently used cell imaging modality.

**Table 14:** A summary of conventional optical imaging modalities reported in the surveyed publications

Imaging Modality	Bright Field	Dark Field	Confocal fluorescence	Wide-Field Fluorescence	DIC	Phase contrast	Two-photon fluorescence	Light sheet
Occurrence	7	1	33	22	4	19	1	1

The output of the segmentation algorithm in most of the works surveyed was in the form of pixels or voxels. Some other works contained algorithms that generated (a) polygons or triangle meshes [87, 89] (b) spherical harmonics [64, 66], or (c) B-splines [65].

### Co-occurrence of segmented objects and evaluation approaches

In terms of segmentation evaluations, Table 15 shows another summary of a co-occurrence of publications that reported segmentation evaluation approaches and segmented objects. The column “Visual Inspection” is for papers reporting visual inspection of segments usually by a biologist. A biologist would assess whether the segmented results are within his/her visual tolerance, without manually segmenting images. The columns “Object-level evaluation” and “Pixel-level evaluation” are for papers where a concrete segmentation accuracy measure was applied against a segmentation reference, usually established by manual segmentation. This reference can have pixel-level information, for instance, manual segmentation of the shape of the object of interest, or object-level information, for instance, object centroid position or cell counts. Sometimes, a visual inspection is also performed as an additional verification. The column “Unknown” is for papers not mentioning a segmentation evaluation for the corresponding segmented object in comparison to the column “techniques is not specified” but results are reported. We also distinguished two types of synthetic objects that can be used for segmentation evaluation in two separate rows: computer-generated digital models and reference material (e.g., beads).

**Table 15:** Summary statistics of pairs of segmented objects and segmentation evaluation approaches based on the surveyed literature

	Unknown	Object-level evaluation	Pixel-level evaluation	Visual inspection	Technique not specified
Cell	16	9	16	10	1
Other	2	1	4	4	0
Nucleus	4	9	5	7	0
Synthetic (digital model)	1	1	6	7	0
Synthetic (reference material)	1	0	0	0	0

### Statistics about segmentation software

We found 51 papers presenting novel techniques that also discussed implementation details, source code, or tool availability. Of these 51 papers, 23 either declared their segmentation code as open source or provided access to it on request. The remaining 28 papers discussed implementation details such as the programming language used, but did not mention code availability. The programming languages used to implement novel segmentation techniques are summarized in Table 16.

**Table 16:** A summary of implementation languages encountered during this literature survey

Programming language	Matlab	C++	Java	C	Matlab with C/C++	R	C++ with IDL
Occurrence	20	9	6	4	4	2	2

MATLAB was occasionally supplemented with compiled C/C++ code. Similarly, C++ was paired with IDL (Interactive Data Language). Implementations in C or C++ were sometimes supported by toolkits or libraries; for example QT, GTK, OpenCV, ITK, and VTK. It is assumed that any publication without reference to a development language used other widely available tools, for example, ImageJ/Fiji.

Of the 72 papers surveyed for computational platforms, most either did not report the hardware on which they were tested or did not explicitly claim support for any sort of parallelism. Of the works that did claim support for parallelism, one ran on a cluster [81], one ran on a GPU [82], and five had explicit support for multi-core CPUs [83–87]. It is possible, however, that even code that was not explicitly designed to support parallelism might still support it, perhaps even unbeknownst to the code’s authors, through the lower-level libraries on which the code is based. For example, Matlab provides built-in multithreading for several functions, such as *fft* and *sort* [92]. This ambiguity may suggest a need for an improved standard in the literature for the reporting of the platform on which algorithms are benchmarked.

## Conclusions

This survey provides information about capabilities and limitations of segmentation techniques that have been applied to cellular measurements of mammalian cells from optical microscopy images. We categorized the types of cellular measurements, segmentation algorithms, segmented objects, segmentation evaluations, and hardware platforms for accelerating image segmentation-based cellular measurements. Occurrence and co-occurrence statistics of published work since 2000 are presented here and on-line. These statistics provide an insight for cell biologists and computer scientists about the choice of a segmentation method, its evaluation approach, and a computational scalability in the context of cellular measurements.

While preparing this survey, we have identified a few open research questions and topics for which future research would need additional documentation [93].

Open research questions:

- One of the frequent questions is: What should I do to segment my images? To reduce the amount of time spent developing new segmentation algorithms for problems that have existing solutions, there is an open problem of designing a recommendation system which can automatically recommend segmentation algorithms based on input information, such as the imaging mode and cellular model system. Such a smart system could lead to a very general image analysis solution pipeline for biologists.
- Segmentation evaluation is an open problem due to the dependency on reference segmentations and its creation process, the lack of sampling considerations during manual segmentation as a reference, and the difficulty in comparing multiple evaluation measures. Reference materials and “cell phantoms” might be useful as a starting point.
- As the imaging measurement pipeline consists of several computations, there is a need to understand the associated uncertainties and to combine them into a “combined standard uncertainty” for the object metrics. There are methods for assessing uncertainty in different parts of the pipeline, but there is not an approach for expressing the uncertainties with the measured object metrics.
- There is an open research question about consolidating terms used in publications. One example is an unclear taxonomy for segmentation evaluations that include simulated data, reference materials such as beads or cell phantoms, manually contoured segments, and manually selected parameters of segmentations.

Topics for which future research needs additional documentation:

- The lack of exploitation of advanced hardware platforms and segmentation parallelism in the surveyed literature opens up the question of whether there are more computationally complex algorithms that might provide higher accuracy.
- While segmentation algorithm speed is increasing in importance, we found it difficult to compare algorithm execution times based on information in publications. Similarly, reference segmentation objects were reported in various “units” and at object- or pixel-levels. This opens a question about introducing a guideline for reporting segmentation evaluations.
- The lack of machine learning based approaches to segmentation in the surveyed literature might suggest that data-driven approaches to segmentation are under explored. With the advances in deep and other learning methods, there is a question whether segmentation of biological objects could be learned from large collections of images.
- There is a need for a number of reference materials to assess and document the performance of various steps in the imaging measurement pipeline
  - ○ Reference optical filters or reference solutions to benchmark microscope performance
  - ○ Reference images of cells that have been analyzed by a consortium and have been assigned measurement tolerances for object metrics
  - ○ Reference slides of 2D cells or 3D scaffolds with cells that have been imaged and analyzed by a consortium, and have been assigned measurement tolerances
  - ○ Reference 3D structures with known geometries that can be imaged and processed to assess performance of the imaging pipeline, especially fluorescent reference spheroids with refractive indices that match cells.

## Endnotes

The papers selected for this survey were targeted to meet the criteria reflected in the survey title. The survey criteria can be described at a high level as follows:

- biological focus: mammalian cells AND
- measurement instrument: optical microscope AND
- cellular measurement: derived from [x, y, time] or [x, y, z] observations AND
- image processing step: automated segmentation.

In order to identify the core set of papers for this survey, we explored a variety of search strings, for the date range 2000–2015, and two databases: Web of Science and PubMed. The example search strings are below:

1. Mammalian cell* AND (2D OR two dimension* OR 2 dimension* OR 3D OR three dimension* OR three dimension*) AND optical AND (phase contrast OR confocal OR Differential Interference contrast OR DIC OR fluorescent OR Selective Plane Illumination Microscopy OR SPIM) AND (design OR segmentation)
  Web of Science Result: 2 records; PubMed Result: 1 record

(2) optical microscopy AND imag* AND segmentation AND cell*
  Web of Science Result: 145 records; PubMed Result: 95 records

(3) optical microscopy AND imag* AND segmentation AND cell* AND (3D OR three dimension* OR 3 dimension* OR tracking)
  Web of Science Result: 80 records; PubMed Result: 50 records

(4) optical microscopy AND imag* AND segmentation AND cell* AND (3D OR three dimension* OR 3 dimension* OR tracking) NOT (MRI OR magnetic resonance imaging OR PET OR positron electron tomography OR CT OR computer tomography)
  Web of Science Result: 72 records; PubMed Result: 48 records

As seen above, our initial definition of the search strings included the key words, such as “optical microscopy”, “segmentation”, “cell”, “three dimensions”, and “mammalian”. The word “mammalian” was eliminated later because many papers focusing on mammalian cells do not use the word explicitly. The words “three dimensions” or 3D were also not specific enough to select papers focusing on segmentation of 3D data including 2D + time and X-Y-Z or time sequences of X-Y-Z (denoted as X-Y-Z-Time). These data types are tacitly assumed in publications while referring to problems, for instance, cell tracking (X-Y-Time or X-Y-Z-Time), or cell-scaffold interaction (X-Y-Z). In many cases segmentation is addressed in one sentence in the methods section. A search of “segmentation” and “3D imaging” is unreliable since “segmentation” is rarely used as an indexing term or mentioned in the title/abstract by cell biologists. We also observed that the search key words “optical microscopy” were sometimes matched with the optical flow technique applied to microscopy images.

In several automated searchers, we also explicitly excluded the key words “magnetic resonance imaging” or “positron electron tomography” or “computer tomography”. These key words are frequently found in the medical imaging domain focusing on segmentation of mammalian organs in conjunction with microscopy image analysis (histopathology). We focused this survey on cell imaging modalities that exclude the above imaging modalities. As a result, we used the above inclusion and exclusion key words for searching but had to manually filter all publications found by the automated search. For this survey, we searched specifically the Web of Science and PubMed databases in addition to the Internet.

We would also like to mention that the segmentation survey is primarily focused on 3D data sets. While the data sets with [x, y, time] dimensions could be segmented in 2D and then post-processed in 3D, we did not consider those papers that focused on 2D segmentation. The reasons lie in the survey focus on cellular measurements derived from 3D data, and the explosion of the number of publications if 2D segmentation would be included. Thus, the topics related to 2D segmentation or cell tracking that perform 2D segmentation independently of the cell correspondence over time are not covered since they would need their own surveys. In the case of object tracking, we included the methods that perform segmentation and object correspondence in tandem since they operate on 3D data sets.

During the manual inspection process, co-authors of this survey went over close to 1000 publications. They decided to include some papers that demonstrate key aspects of segmentation although the measurements were not applied to cells, as well as to exclude some papers that use less frequently used cell imaging modalities than phase contrast or DIC or confocal laser scanning or fluorescent or dark/bright field modality. Many co-authors followed chains of related papers. The assumption is that a high quality paper will cite many other papers relevant to the topic. Following these citation links often finds relevant new papers that a search missed. Unfortunately, while this approach produces useful papers, it does not allow for an algorithmic definition of the paper search.

## Availability of supporting data

The spread sheet with the literature classification is available on-line at https://isg.nist.gov/deepzoomweb/resources/survey/SegmSurvey_classifications.txt.

The histogram and co-occurrence tables at https://isg.nist.gov/deepzoomweb/resources/survey/index.html have hyperlinks to web pages that show the list of publications contributing to each statistical entry.

## References

[1] Watson P (2009). Live cell imaging for target and drug discovery. Drug News Perspect.

[2] Brown GC, Brown MM, Sharma S, Stein JD, Roth Z, Campanella J (2005). The burden of age-related macular degeneration: a value-based medicine analysis. Trans Am Ophthalmol Soc.

[3] Branstetter BF, Faix LE, Humphrey A, Schumann J (2007). Preclinical medical student training in radiology: the effect of early exposure. Am J Roentgenol (AJR).

[4] Swedlow JR, Goldberg I, Brauner E, Sorger PK (2003). Informatics and quantitative analysis in biological imaging. Science (New York, NY).

[5] Cell Stains [http://www.lifetechnologies.com/order/catalog/en/US/adirect/lt?cmd=IVGNcatDisplayCategory&catKey=68901)].

[6] Glanzel W, Schubert A (2003). A new classification scheme of science fields and subfields designed for scientometric evaluation purposes. Scientometrics.

[7] Wirjadi O. Report: Survey of 3D Image Segmentation Methods. Fraunhofer-Institut für Techno- undWirtschaftsmathematik, Kaiserslautern, Germany, 2007: 1-29; ISSN 1434-9973; https://kluedo.ub.unikl.de/files/1978/bericht123.pdf; Last time accessed: 10-12-2015.

[8] Kalinic H. Report: Atlas-Based Image Segmentation: A Survey. University of Zagreb, Zagreb, Croatia; 2008:1–7. http://bib.irb.hr/datoteka/435355.jnrl.pdf; Last time accessed: 10-12-2015.

[9] Zhang Y (1996). A survey on evaluation methods for image segmentation. Pattern Recogn.

[10] Dima AA, Elliott JT, Filliben JJ, Halter M, Peskin A, Bernal J (2011). Comparison of segmentation algorithms for fluorescence microscopy images of cells. Cytometry Part A: the journal of the International Society for Analytical Cytology.

[11] Bhadriraju K, Elliott JT, Nguyen M, Plant AL (2007). Quantifying myosin light chain phosphorylation in single adherent cells with automated fluorescence microscopy. BMC Cell Biol.

[12] North AJ (2006). Seeing is believing? A beginners’ guide to practical pitfalls in image acquisition. J Cell Biol.

[13] Dolman NJ, Kilgore JA, Davidson MW (2013). A review of reagents for fluorescence microscopy of cellular compartments and structures, part I: BacMam labeling and reagents for vesicular structures. Current protocols in cytometry / editorial board, J Paul Robinson, managing editor [et al].

[14] Kilgore JA, Dolman NJ, Davidson MW (2013). A review of reagents for fluorescence microscopy of cellular compartments and structures, Part II: reagents for non-vesicular organelles. Current protocols in cytometry / editorial board, J Paul Robinson, managing editor [et al].

[15] Kilgore JA, Dolman NJ, Davidson MW (2014). A review of reagents for fluorescence microscopy of cellular compartments and structures, Part III: reagents for actin, tubulin, cellular membranes, and whole cell and cytoplasm. Current protocols in cytometry / editorial board, J Paul Robinson, managing editor [et al].

[16] Giuliano K, Taylor D, Waggoner A. Reagents to measure and manipulate cell functions. In Methods in Molecular Biology. Volume 356. Edited by D. L. Taylor, J. R. Haskins and KG. Humana Press, Inc.; 2006:141–162. 10.1385/1-59745-217-3:141 16988401

[17] Niles WD, Coassin PJ (2008). Cyclic olefin polymers: innovative materials for high-density multiwell plates. Assay Drug Dev Technol.

[18] Buchser W, Collins M, Garyantes T, Guha R, Haney S, Lemmon V, Sittampalam G, Coussens N, Nelson H (2014). Assay Development Guidelines for Image-Based High Content Screening, High Content Analysis and High Content Imaging. Assay Guidance Manual. Volume Intenet.

[19] Murphy WL, McDevitt TC, Engler AJ (2014). Materials as stem cell regulators. Nat Mater.

[20] Murphy D (2001). Fundamentals of Light Microscopy and Electronic Imaging.

[21] Pawley J (2006). Handbook of Biological Confocal Microscopy.

[22] Frigault MM, Lacoste J, Swift JL, Brown CM (2009). Live-cell microscopy - tips and tools. J Cell Sci.

[23] Plant AL, Elliott JT, Bhat TN (2011). New concepts for building vocabulary for cell image ontologies. BMC Bioinformatics.

[24] Khan I, Fraser A, Bray M-A, Smith PJ, White NS, Carpenter AE (2014). ProtocolNavigator: emulation-based software for the design, documentation and reproduction biological experiments. Bioinformatics (Oxford, England).

[25] Goldberg IG, Allan C, Burel J-M, Creager D, Falconi A, Hochheiser H (2005). The Open Microscopy Environment (OME) Data Model and XML file: open tools for informatics and quantitative analysis in biological imaging. Genome Biol.

[26] Eliceiri KW, Berthold MR, Goldberg IG, Ibáñez L, Manjunath BS, Martone ME (2012). Biological imaging software tools. Nat Methods.

[27] Begley CG, Ellis LM (2012). Drug development: raise standards for preclinical cancer research. Nature.

[28] Mobley A, Linder S, Braeuer R, Ellis L, Zwelling L (2013). A survey on data reproducibility in cancer research provides insights into our limited ability to translate findings from the laboratory to the clinic. PLoS One.

[29] Chang HH, Hemberg M, Barahona M, Ingber DE, Huang S (2008). Transcriptome-wide noise controls lineage choice in mammalian progenitor cells. Nature.

[30] Singer ZS, Yong J, Tischler J, Hackett JA, Altinok A, Surani MA (2014). Dynamic heterogeneity and DNA methylation in embryonic stem cells. Mol Cell.

[31] Sigal A, Milo R, Cohen A, Geva-Zatorsky N, Klein Y, Alaluf I (2006). Dynamic proteomics in individual human cells uncovers widespread cell-cycle dependence of nuclear proteins. Nat Methods.

[32] Elliott JT, Tona A, Woodward JT, Jones PL, Plant AL (2003). Thin films of collagen affect smooth muscle cell morphology. Langmuir.

[33] Halter M, Tona A, Bhadriraju K, Plant AL, Elliott JT (2007). Automated live cell imaging of green fluorescent protein degradation in individual fibroblasts. Cytometry Part A : the journal of the International Society for Analytical Cytology.

[34] Ravin R, Hoeppner DJ, Munno DM, Carmel L, Sullivan J, Levitt DL (2008). Potency and fate specification in CNS stem cell populations *in vitro*. Cell Stem Cell.

[35] Cukierman E, Pankov R, Stevens DR, Yamada KM (2001). Taking cell-matrix adhesions to the third dimension. Science (New York, NY).

[36] Blatter LA (1999). Cell volume measurements by fluorescence confocal microscopy: theoretical and practical aspects. Methods Enzymology.

[37] Shaw M, Faruqui N, Gurdak E, Tomlins P (2013). Three-dimensional cell morphometry for the quantification of cell-substrate interactions. Tissue engineering Part C, Methods.

[38] Liang XJ, Liu AQ, Lim CS, Ayi TC, Yap PH (2007). Determining refractive index of single living cell using an integrated microchip. Sensors Actuators A Phys.

[39] Chaitavon K, Sumriddetchkajorn S, Nukeaw J (2013). Highly sensitive refractive index measurement with a sandwiched single-flow-channel microfluidic chip. RSC Advances.

[40] LaFratta CN, Fourkas JT, Baldacchini T, Farrer RA (2007). Multiphoton fabrication. Angewandte Chemie (International ed in English).

[41] Vantaram SR, Saber E (2012). Survey of contemporary trends in color image segmentation. J Electronic Imaging.

[42] Al-Kofahi O, Radke RJ, Goderie SK, Shen Q, Temple S, Roysam B (2006). Automated cell lineage construction: a rapid method to analyze clonal development established with murine neural progenitor cells. Cell cycle (Georgetown, Tex).

[43] Otsu N (1979). A tlreshold selection method from gray-level histograms. IEEE Transactions on Systems, Man and Cybernetics.

[44] Bise R, Li K, Eom S. Reliably tracking partially overlapping neural stem cells in dic microscopy image sequences. In MICCAI Workshop on Optical Tissue Image analysis in Microscopy, Histopathology and Endoscopy. Imperial College London; 2009:67–77.

[45] Way D (2006). Principles and applications of differential interference contrast light microscopy. Microscopy and Analysis; Light Microscopy Supplement.

[46] Ewers H, Smith AE, Sbalzarini IF, Lilie H, Koumoutsakos P, Helenius A (2005). Single-particle tracking of murine polyoma virus-like particles on live cells and artificial membranes. Proc Natl Acad Sci U S A.

[47] Gordon A, Colman-Lerner A, Chin TE, Benjamin KR, Yu RC, Brent R. Single-cell quantification of molecules and rates using open-source microscope-based cytometry. Nat Methods. 2007;175–181. 10.1038/nmeth1008 17237792

[48] Hadjidemetriou S, Gabrielli B, Mele K, Vallotton P (2008). Detection and tracking of cell divisions in phase contrast video microscopy. Proc. of the Third MICCAI Worshop on Microscopic Image Analysis with Applications in Biology.

[49] Kerschnitzki M, Kollmannsberger P, Burghammer M, Duda GN, Weinkamer R, Wagermaier W (2013). Architecture of the osteocyte network correlates with bone material quality. J Bone Miner Res.

[50] Pérez De Alejo R, Ruiz-Cabello J, Cortijo M, Rodriguez I, Echave I, Regadera J (2003). Computer-assisted enhanced volumetric segmentation magnetic resonance imaging data using a mixture of artificial neural networks. Magn Reson Imaging.

[51] Dickens MM, Gleason SS, Sari-Sarraf H (2002). Volumetric segmentation via 3D active shape models. Proceedings of the Fifth IEEE Southwest Symposium on Image Analysis and Interpretation.

[52] Zahalka A, Fenster A (2001). An automated segmentation method for three-dimensional carotid ultrasound images. Phys Med Biol.

[53] Zhang H, Fritts JE, Goldman SA (2008). Image segmentation evaluation: a survey of unsupervised methods. Comput Vis Image Underst.

[54] Lin G, Chawla MK, Olson K, Barnes CA, Guzowski JF, Bjornsson C (2007). A multi-model approach to simultaneous segmentation and classification of heterogeneous populations of cell nuclei in 3D confocal microscope images. Cytometry Part A.

[55] Lin G, Adiga U, Olson K, Guzowski JF, Barnes CA, Roysam B (2003). A hybrid 3D watershed algorithm incorporating gradient cues and object models for automatic segmentation of nuclei in confocal image stacks. Cytometry Part A: the journal of the International Society for Analytical Cytology.

[56] Padfield D, Rittscher J, Roysam B (2008). Spatio-temporal cell segmentation and tracking for automated screening. Processings on the 5th IEEE International Symposium on Biomedical Imaging: From Nano to Macro.

[57] Krzic U, Gunther S, Saunders TE, Streichan SJ, Hufnagel L (2012). Multiview light-sheet microscope for rapid *in toto* imaging. Nat Methods.

[58] Gelasca ED, Obara B, Fedorov D, Kvilekval K, Manjunath B (2009). A biosegmentation benchmark for evaluation of bioimage analysis methods. BMC Bioinformatics.

[59] Martone ME, Tran J, Wong WW, Sargis J, Fong L, Larson S (2008). The cell centered database project: an update on building community resources for managing and sharing 3D imaging data. J Struct Biol.

[60] Blackshaw S, Harpavat S, Trimarchi J, Cai L, Huang H, Kuo WP (2004). Genomic analysis of mouse retinal development. PLoS Biol.

[61] Singh S, Raman S, Rittscher J, Machiraju R (2009). Segmentation Evaluation for Fluorescence Microscopy Images of Biological Objects. MIAAB 2009 International Workshop Proceedings.

[62] Peskin A, Chalfoun J, Kafadar K, Elliott J (2013). Estimating the Number of Manually Segmented Cellular Objects Required to Evaluate the Accuracy of a Segmentation Algorithm. Proceedings of ACM BCB.

[63] Wählby C, Sintorn I-M, Erlandsson F, Borgefors G, Bengtsson E (2004). Combining intensity, edge and shape information for 2D and 3D segmentation of cell nuclei in tissue sections. J Microsc.

[64] Du C, Hawkins P (2013). 3D time series analysis of cell shape using Laplacian approaches. BMC Bioinformatics.

[65] Delgado-Gonzalo R, Chenouard N, Unser M (2013). Spline-based deforming ellipsoids for interactive 3D bioimage segmentation. IEEE Trans Image Process.

[66] Eck S, Rohr K, Biesdorf A, Katharina M-O, Rippe K, Stefan W (2013). A 3D Intensity Model Based on Spherical Harmonics For Automatic 3D Segmentation of Heterochromatin Foci.

[67] Hodneland E, Bukoreshtliev NV, Eichler TW, Tai X-C, Gurke S, Lundervold A (2009). A unified framework for automated 3-d segmentation of surface-stained living cells and a comprehensive segmentation evaluation. IEEE Trans Med Imaging.

[68] Dufour A, Thibeaux R, Labruyère E, Guillén N, Olivo-Marin J-C (2011). 3-D active meshes: fast discrete deformable models for cell tracking in 3-D time-lapse microscopy. IEEE Trans Image Process.

[69] Dzyubachyk O, Van Cappellen WA, Essers J, Niessen WJ, Meijering E (2010). Advanced level-set-based cell tracking in time-lapse fluorescence microscopy. IEEE Trans Med Imaging.

[70] Kriston-Vizi J, Thong NW, Poh CL, Yee KC, Ling JSP, Kraut R (2011). Gebiss: an ImageJ plugin for the specification of ground truth and the performance evaluation of 3D segmentation algorithms. BMC Bioinformatics.

[71] Deza MM, Deza E (2013). Encyclopedia of Distances.

[72] Stegmaier J, Otte JC, Kobitski A, Bartschat A, Garcia A, Nienhaus GU (2014). Fast segmentation of stained nuclei in terabyte-scale, time resolved 3D microscopy image stacks. PLoS One.

[73] Takemoto S, Yokota H. Algorithm selection based on a region similarity metric for intracellular image segmentation. In Image Segmentation. Edited by Dr Ho P-G. InTech; 2011:419–434.

[74] Maška M, Ulman V, Svoboda D, Matula P, Matula P, Ederra C (2014). A benchmark for comparison of cell tracking algorithms. Bioinformatics.

[75] Bajcsy P, Simon M, Florczyk S, Simon C, Juba D, Brady M. A Method for the Evaluation of Thousands of Automated 3D Stem Cell Segmentations. Journal of Microscopy 2015:under review. 10.1111/jmi.12303 PMC4888372 26268699

[76] Lu Z, Carneiro G, Bradley A (2013). Automated nucleus and cytoplasm segmentation of overlapping cervical cells. Medical Image Computing and Computer-Assisted Intervention (MICCAI). Volume 8149.

[77] Plissiti ME, Nikou C (2012). Overlapping cell nuclei segmentation using a spatially adaptive active physical model. IEEE Trans Image Process.

[78] Schindelin J, Arganda-Carreras I, Frise E, Kaynig V, Longair M, Pietzsch T (2012). Fiji: an open-source platform for biological-image analysis. Nat Methods.

[79] Schnabel R, Hutter H, Moerman D, Schnabel H. Assessing Normal Embryogenesis in Caenorhabditis elegans Using a 4D Microscope : Variability of Development and Regional Specification. 1997, 265:234–265. 10.1006/dbio.1997.8509 9133433

[80] Shi L, Liu W, Zhang H, Xie Y, Wang D (2012). A survey of GPU-based medical image computing techniques. Quantitative Imaging Med Surgery.

[81] Buggenthin F, Marr C, Schwarzfischer M, Hoppe PS, Hilsenbeck O, Schroeder T (2013). An automatic method for robust and fast cell detection in bright field images from high-throughput microscopy. BMC Bioinformatics.

[82] Juba D, Cardone A, Ip CY, Simon Jr CG, Tison CK, Kumar G, et al. Parallel geometric classification of stem cells by their 3D morphology. Computational Science Discovery. 2013;6.

[83] Mange R, de Heras Ciechomski P, Swartz M (2008). seeCell: Visualization and tracking dedicated to cell analysis. 2008 International Conference on Innovations in Information Technology.

[84] Lou X, Kang M, Xenopoulos P, Muñoz-Descalzo S, Hadjantonakis A-K (2014). A rapid and efficient 2D/3D nuclear segmentation method for analysis of early mouse embryo and stem cell image data. Stem Cell Reports.

[85] Krausz E, de Hoogt R, Gustin E, Cornelissen F, Grand-Perret T, Janssen L, et al. Translation of a Tumor Microenvironment Mimicking 3D Tumor Growth Co-culture Assay Platform to High-Content Screening. Journal of Biomolecular Screening 2012. 10.1177/1087057112456874 22923784

[86] Celli JP, Rizvi I, Evans CL, Abu-Yousif AO, Hasan T (2010). Quantitative imaging reveals heterogeneous growth dynamics and treatment-dependent residual tumor distributions in a three-dimensional ovarian cancer model. J Biomed Opt.

[87] Winter M, Wait E, Roysam B, Goderie SK, Ali RAN, Kokovay E (2011). Vertebrate neural stem cell segmentation, tracking and lineaging with validation and editing. Nat Protoc.

[88] Cohen AR, Gomes FLAF, Roysam B, Cayouette M (2010). Computational prediction of neural progenitor cell fates. Nat Methods.

[89] Pop S, Dufour A, Le GJ, Ragni CV, Buckingham ME, Meilhac SM (2011). A Fast and Automated Framework for Extraction of Nuclei From Cluttered 3D Images in Fluorescence Microscopy. 2011 IEEE International Symposium on Biomedical Imaging: From Nano to Macro.

[90] Sezgin M, Sankur B (2004). Survey over image thresholding techniques and. J Electronic Imaging.

[91] Padfield DR, Rittscher J, Sebastian T, Thomas N, Roysam B (2006). Spatio-Temporal Cell Cycle Analysis Using 3D Level Set Segmentation of Unstained Nuclei in Line Scan Confocal Fluorescence Images. 3rd IEEE International Symposium on Biomedical Imaging: Macro to Nano.

[92] MATLAB Multicore [http://www.mathworks.com/discovery/matlab-multicore.html].

[93] Trikalinos T, Dahabreh I, Lee J. Methods research on future research needs: defining an optimal format for presenting research needs. Methods Future Res Needs Report. 2011;1–43. 21977525

[94] ASTM (2014). Guide for Using Fluorescence Microscopy to Quantify the Spread Area of Fixed Cells 1.

[95] Song L, Hennink E, Young I, Tanke H (1995). Photobleaching kinetics of fluorescein in quantitative fluorescent microscopy. Biophys J.

[96] Antibody Validation Criteria [http://www.antibodypedia.com/text/validation_criteria].

[97] Bordeaux J, Welsh A, Agarwal S, Killiam E, Baquero M, Hanna J (2010). Antibody validation. BioTechniques.

[98] Begley CG (2013). Six red flags for suspect work. Nature.

[99] Neumann M, Gabel D (2002). Simple method for reduction of autofluorescence in fluorescence microscopy. J Histochemistry Cytochemistry.

[100] Andersson H, Baechi T, Hoechl M, Richter C (1998). Autofluorescence of living cells. J Microsc.

[101] Autofluorescence: Causes and cures http://www.uhnres.utoronto.ca/facilities/wcif/PDF/Autofluorescence.pdf.

[102] Kennedy SB, Washburn NR, Simon CG, Amis EJ (2006). Combinatorial screen of the effect of surface energy on fibronectin-mediated osteoblast adhesion, spreading and proliferation. Biomaterials.

[103] Hng KI, Dormann D (2013). ConfocalCheck--a software tool for the automated monitoring of confocal microscope performance. PLoS One.

[104] Halter M, Bier E, Derose PC, Cooksey GA, Choquette SJ, Plant AL (2014). An automated protocol for performance benchmarking a widefield fluorescence microscope.

[105] Matching Fluorescent Probes With Nikon Fluorescence Filter Blocks [http://microscopyu.com/tutorials/flash/spectralprofiles/index.html].

[106] Cole RW, Jinadasa T, Brown CM (2011). Measuring and interpreting point spread functions to determine confocal microscope resolution and ensure quality control. Nat Protoc.

[107] NIST (2009). Report of Investigation: Scanning Electron Microscope Scale Calibration Artifact Reference.

[108] Jensen KE, Weitz DA, Spaepen F (2013). Note: a three-dimensional calibration device for the confocal microscope. Rev Sci Instrum.

[109] Benson DM, Bryan J, Plant AL, Gotto AM, Smith LC (1985). Digital imaging fluorescence microscopy : spatial heterogeneity of photobleaching rate constants in individual cells. J Cell Biol.

[110] Model MA, Burkhardt JK (2001). A standard for calibration and shading correction of a fluorescence microscope. Cytometry.

[111] Young IT (2000). Shading correction : compensation for illumination and sensor inhomogeneities. Current Protocols in Cytometry.

[112] Zwier JM, Van Rooij GJ, Hofstraat JW, Brakenhoff GJ (2004). Image calibration in fluorescence microscopy. J Microsc.

[113] Park DY, Jones D, Moldovan NI, Machiraju R, Pecot T (2013). Robust detection and visualization of cytoskeletal structures in fibrillar scaffolds from 3-dimensional confocal image. IEEE Symposium on Biological Data Visualization (BioVis).

[114] Grosse R, Vartiainen MK (2013). To be or not to be assembled: progressing into nuclear actin filaments. Nat Rev Mol Cell Biol.

[115] Sbalzarini IF, Koumoutsakos P (2005). Feature point tracking and trajectory analysis for video imaging in cell biology. J Struct Biol.

[116] Bajcsy P, Lee S-C, Lin A, Folberg R (2006). Three-dimensional volume reconstruction of extracellular matrix proteins in uveal melanoma from fluorescent confocal laser scanning microscope images. J Microsc.

[117] Herberich G, Windoffer R, Leube R, Aach T (2011). 3D segmentation of keratin intermediate filaments in confocal laser scanning microscopy. Annual International Conference of the IEEE Engineering in Medicine and Biology Society.

[118] Bai W, Zhou X, Zhu J, Ji L, Wong STC (2007). Tracking of migrating Glioma cells in feature space. 4th IEEE International Symposium on Biomedical Imaging: From Nano to Macro. IEEE.

[119] Huh S, Eom S, Bise R, Yin Z, Kanade T (2011). Mitosis detection for stem cell tracking in phase-contrast microscopy images. Biomedical Imaging.

[120] Chalfoun J, Kociolek M, Dima A, Halter M, Cardone A, Peskin A (2013). Segmenting time-lapse phase contrast images of adjacent NIH 3 T3 cells. J Microsc.

[121] Chalfoun J, Cardone A, Dima A (2010). Overlap-based cell tracker. J Res National Institute Standards Technol.

[122] Chalfoun J, Majurski M, Dima A, Stuelten C, Peskin A (2014). FogBank: a single cell segmentation across multiple cell lines and image modalities. BMC Bioinformatics.

[123] Indhumathi C, Cai YY, Guan YQ, Opas M (2011). An automatic segmentation algorithm for 3D cell cluster splitting using volumetric confocal images. J Microsc.

[124] Wang Q, Niemi J, Tan C-M, You L, West M (2010). Image segmentation and dynamic lineage analysis in single-cell fluorescence microscopy. Cytometry Part A: the journal of the International Society for Analytical Cytology.

[125] Yu W, Lee HK, Hariharan S, Bu W, Ahmed S (2010). Evolving generalized Voronoi diagrams for accurate cellular image segmentation. Cytometry Part A: the journal of the International Society for Analytical Cytology.

[126] Chinta R, Wasser M (2012). Three-dimensional segmentation of nuclei and mitotic chromosomes for the study of cell divisions in live Drosophila embryos. Cytometry Part A: the journal of the International Society for Analytical Cytology.

[127] Li K, Miller ED, Chen M, Kanade T, Weiss LE, Campbell PG (2008). Cell population tracking and lineage construction with spatiotemporal context. Med Image Anal.

[128] Chalfoun J, Majurski M, Bhadriraju K, Lund S, Bajcsy P, Brady M (2015). Background intensity correction for terabyte-sized time-lapse images. J Microsc.

[129] Chalfoun J, Majurski M, Peskin A, Breen C, Bajcsy P. Empirical gradient threshold technique for automated segmentation across image modalities and cell lines. J Microsc. 2014;1–18. 10.1111/jmi.12269 26046924

[130] Li K, Miller ED, Weiss LE, Campbell PG, Kanade T. Online Tracking of Migrating and Proliferating Cells Imaged with Phase-Contrast Microscopy. In: 2006 Conference on Computer Vision and Pattern Recognition Workshop (CVPRW’06). 2006. p. 65–5.

[131] Srinivasa G, Fickus M, Kovačević J, Van De Ville D, Goyal VK, Papadakis M (2007). Active Contour-Based Multiresolution Transforms for the Segmentation of Fluorescence Microscope Images. Proceedings of the SPIE.

[132] Fehr J, Ronneberger O, Kurz H, Burkhardt H, Kropatsch W, Sablating R (2005). Self-learning Segmentation and Classification of Cell-Nuclei in 3D Volumetric Data Using Voxel-Wise Gray Scale Invariants. Pattern Recognition.

[133] Srinivasa G, Fickus MC, Guo Y, Linstedt AD, Kovacević J (2009). Active mask segmentation of fluorescence microscope images. IEEE Trans Image Process.

[134] Peng T, Murphy RF (2011). Image-derived, three-dimensional generative models of cellular organization. Cytometry Part A : the journal of the International Society for Analytical Cytology.

[135] Sommer C, Straehle C (2011). Ilastik: Interactive learning and segmentation toolkit. 2011 IEEE International Symposium on Biomedical Imaging: From Nano to Macro.

[136] Bjornsson C, Lin G, Al-Kofahi Y, Narayanaswamy A, Smith KL, Shain W (2008). Associative image analysis: a method for automated quantification of 3D multi-parameter images of brain tissue. J Neuroscience Methods Methods.

[137] Yoo TS, Ackerman MJ, Lorensen WE, Schroeder W, Chalana V, Aylward S (2002). Engineering and algorithm design for an image processing Api: a technical report on ITK--the Insight Toolkit. Stud Health Technol Inform.

[138] Mosaliganti K, Cooper L (2008). Reconstruction of cellular biological structures from optical microscopy data. IEEE Trans Vis Comput Graph.

[139] Li G, Liu T, Tarokh A, Nie J, Guo L, Mara A (2007). 3D cell nuclei segmentation based on gradient flow tracking. BMC Cell Biol.

[140] Peng H, Ruan Z, Long F, Simpson JH, Myers EW (2010). V3D enables real-time 3D visualization and quantitative analysis of large-scale biological image data sets. Nat Biotechnol.

[141] Hodneland E, Kögel T, Frei DM, Gerdes H-H, Lundervold A. Cell Segm - a MATLAB toolbox for highthroughput3D cell segmentation. Source Code Biol Med., BioMed Central Ltd.; 2013;8:16. doi:10.1186/1751-0473-8-16 Last time accessed: 10-12-2015. 10.1186/1751-0473-8-16 PMC3850890 23938087

[142] Andrey P, Maurin Y (2005). Free-D: an integrated environment for three-dimensional reconstruction from serial sections. J Neurosci Methods.

[143] Long F, Peng H, Liu X, Kim SK, Myers E (2009). A 3D digital atlas of C. elegans and its application to single-cell analyses. Nat Methods.

[144] Carpenter AE, Jones TR. Cell Profiler: image analysis software for identifying and quantifying cell phenotypes. Genome Biol. 2006;7. 10.1186/gb-2006-7-10-r100 PMC1794559 17076895

[145] Kaynig V, Fischer B, Müller E, Buhmann JM (2010). Fully automatic stitching and distortion correction of transmission electron microscope images. J Struct Biol.

[146] Berthold M, Cebron N, Dill F, Gabriel T, Kötter T, Meinl T, Preisach C, Burkhardt H, Schmidt-Thieme L, Decker R (2008). KNIME: The Konstanz Information Miner. Data Analysis, Machine Learning and Applications SE - 38.

[147] Wait E, Winter M, Bjornsson C, Kokovay E, Wang Y, Goderie S (2014). Visualization and correction of automated segmentation, tracking and lineaging from 5-D stem cell image sequences. BMC Bioinformatics.

[148] Allan C, Burel J-M, Moore J, Blackburn C, Linkert M, Loynton S (2012). OMERO: flexible, model-driven data management for experimental biology. Nat Methods.

[149] Edelstein A, Amodaj N, Hoover K, Vale R, Stuurman N, Ausubel FM (2010). Computer control of microscopes using μManager. Current protocols in molecular biology.

[150] Himeno-Ando A, Izumi Y, Yamaguchi A, Iimura T (2012). Structural differences in the osteocyte network between the calvaria and long bone revealed by three-dimensional fluorescence morphometry, possibly reflecting distinct mechano-adaptations and sensitivities. Biochem Biophys Res Commun.

[151] Moeller M, Burger M, Dieterich P, Schwab A (2014). A framework for automated cell tracking in phase contrast microscopic videos based on normal velocities. J Vis Commun Image Represent.

[152] Held M, Schmitz MHA, Fischer B, Walter T, Neumann B, Olma MH (2010). Cell Cognition: time-resolved phenotype annotation in high-throughput live cell imaging. Nat Methods.

[153] Systems Science of Biological Dynamics (SSBD) database [http://ssbd.qbic.riken.jp/].

[154] Yang X, Padfield D (2014). Wavelet-initialized 3D level-set cell segmentation with local background support. Biomedical Imaging (ISBI), 2014 IEEE 11th International Symposium on.

[155] Du C-J, Hawkins PT, Stephens LR, Bretschneider T (2013). 3D time series analysis of cell shape using Laplacian approaches. BMC Bioinformatics.

[156] Russell RA, Adams NM, Stephens DA, Batty E, Jensen K, Freemont PS (2009). Segmentation of fluorescence microscopy images for quantitative analysis of cell nuclear architecture. Biophys J.

[157] Yin Z, Li K, Kanade T, Chen M (2010). Understanding the Optics to Aid Microscopy Image Segmentation. Medical Image Computing and Computer-Assisted Intervention--MICCAI 2010.

[158] Yin Z, Kanade T, Chen M (2012). Understanding the phase contrast optics to restore artifact-free microscopy images for segmentation. Med Image Anal.

[159] McCullough DP, Gudla PR, Harris BS, Collins JA, Meaburn KJ, Nakaya MA (2008). Segmentation of whole cells and cell nuclei from 3-D optical microscope images using dynamic programming. IEEE Trans Med Imaging.

[160] Bengtsson E (2004). Combining intensity, edge and shape information for 2D and 3D segmentation of cell nuclei in tissue sections. J Microsc.

[161] Chowdhury S, Ruusuvuori P, Liberali P. Automated cell tracking and cell lineage construction with improved performance. In Proceedings of the Sixth International Workshop on Computational Systems Biology (WCSB). Edited by Manninen T et al. Aarhus, Denmark; 2009:2–5.

[162] Hua J, Sima C, Cypert M, Gooden GC, Shack S, Alla L (2012). Tracking transcriptional activities with high-content epifluorescent imaging. J Biomed Opt.

[163] Hand AJ, Sun T, Barber DC, Hose DR, MacNeil S (2009). Automated tracking of migrating cells in phase-contrast video microscopy sequences using image registration. J Microsc.

[164] Wang M, Zhou X, Li F, Huckins J, King RW, Wong STC (2008). Novel cell segmentation and online SVM for cell cycle phase identification in automated microscopy. Bioinformatics.

[165] Huth J, Buchholz M, Kraus JM, Schmucker M, von Wichert G, Krndija D (2010). Significantly improved precision of cell migration analysis in time-lapse video microscopy through use of a fully automated tracking system. BMC Cell Biol.

[166] Bahnson A, Athanassiou C, Koebler D, Qian L, Shun T, Shields D (2005). Automated measurement of cell motility and proliferation. BMC Cell Biol.

[167] Zhu Y, Olson E, Subramanian A, Feiglin D, Varshney PK, Krol A, Reinhardt JM, Pluim JPW (2008). Neuronal nuclei localization in 3D using level set and watershed segmentation from laser scanning microscopy images. Proc. SPIE. Volume 6914.

[168] Debeir O, Camby I, Kiss R, Van Ham P, Decaestecker C (2004). A model-based approach for automated *in vitro* cell tracking and chemotaxis analyses. Cytometry Part A : the journal of the International Society for Analytical Cytology.

[169] Duncan R. A survey of parallel computer architecture. Computer. 1990;5–16.

